# Novel STAT3 Inhibitors Targeting STAT3 Dimerization by Binding to the STAT3 SH2 Domain

**DOI:** 10.3389/fphar.2022.836724

**Published:** 2022-05-27

**Authors:** Yaping Hua, Xing Yuan, Yun-heng Shen, Jinxin Wang, Waqas Azeem, Shuo Yang, Alexandra Gade, Seyed Mohammad Lellahi, Anne Margrete Øyan, Xisong Ke, Wei-dong Zhang, Karl-Henning Kalland

**Affiliations:** ^1^ Centre for Cancer Biomarkers (CCBIO), Department of Clinical Science, University of Bergen, Bergen, Norway; ^2^ Department of Phytochemistry, School of Pharmacy, Second Military Medical University, Shanghai, China; ^3^ Department of Microbiology, Haukeland University Hospital, Helse Bergen, Bergen, Norway; ^4^ Centre for Cancer Biomarkers, University of Bergen, Bergen, Norway; ^5^ School of Pharmacy, East China University of Science and Technology, Shanghai, China; ^6^ Centre for Molecular Medicine Norway, Faculty of Medicine, University of Oslo, Oslo, Norway; ^7^ Institute of Interdisciplinary Integrative Medicine Research, Shanghai University of Traditional Chinese Medicine, Shanghai, China; ^8^ Department of Immunology and Transfusion Medicine, Haukeland University Hospital, Bergen, Norway

**Keywords:** delavatine A, STAT3, SH2 domain, dimerization, prostate cancer

## Abstract

Our drug discovery model has identified two novel STAT3 SH2 domain inhibitors 323–1 and 323–2 (delavatine A stereoisomers) in a series of experiments. *In silico* computational modeling, drug affinity responsive target stability (DARTS), and fluorescence polarization (FP) assays altogether determined that 323–1 and 323–2 directly target the STAT3 SH2 domain and inhibited both phosphorylated and non-phosphorylated STAT3 dimerization. Computational docking predicted that compound 323s bind to three subpockets of the STAT3 SH2 domain. The 323s inhibition of STAT3 dimerization was more potent than the commercial STAT3 SH2 domain inhibitor S3I-201 in the co-immunoprecipitation assay, correlating with computational docking data. The fluorescence polarization assay further confirmed that the compound 323s target the STAT3 SH2 domain by competitively abrogating the interaction between STAT3 and the SH2-binding peptide GpYLPQTV. Compared with S3I-201, the 323 compounds exhibited stronger inhibition of STAT3 and reduced the level of IL-6-stimulated phosphorylation of STAT3 (Tyr705) in LNCaP cells over the phosphorylation of STAT1 (Tyr701) induced by IFN-ɣ in PC3 cells or the phosphorylation of STAT1 (Ser727) in DU145 cells. Both compounds downregulated STAT3 target genes MCL1 and cyclin D1. Thus, the two compounds are promising lead compounds for the treatment of cancers with hyper-activated STAT3.

## Introduction

The STAT3 pathway is vital to drive PCa progression to metastatic castration-resistant prostate cancer (mCRPC) and integrates with other signaling pathways to activate the androgen receptor (AR) pathway. STAT3 may promote stem-like cells and the epithelial-to-mesenchymal transition (EMT) and interactions between tumor cells and the microenvironment (Bishop et al., 2014). Liu C. et al. (2014) reported that constitutively active STAT3 induced resistance to the androgen receptor inhibitor enzalutamide, and the JAK2 inhibitor AG490 could reverse enzalutamide resistance in LNCaP cells. The level of phospho-STAT3 (pSTAT3 Tyr705) correlates with the pathologic stage, Gleason score, and extracapsular extension in prostate cancer (Horinaga et al., 2005; Liu et al., 2012; Bosch-Barrera and Menendez, 2015). Junaid et *al.* found activation of STAT3 in 67% of bone and 77% of lymph node metastases of prostate cancer patients (Abdulghani et al., 2008). Don-Doncow also found that pSTAT3 was highly expressed in bone metastases, lymph node, and visceral metastases of CRPC patients (Don-Doncow et al., 2016). All these observations suggest that the IL-6/STAT3 pathway promotes tumorigenesis, progression, and metastasis (Pencik et al., 2015a; Pencik et al., 2015b) and may serve as a good target for the treatment of prostate cancer.

STAT3 was originally described in 1993 as a transcription factor in IL-6-stimulated human hepatoma (HepG2) cells (Wegenka et al., 1993; Levy and Lee, 2002; Aigner et al., 2018). The full-length STAT3 has six different structural motifs: a transactivation domain (TAD) for co-factor recruitment, an Src Homology 2 (SH2) domain for receptor binding and dimerization, a linker domain (LD), a DNA-binding domain (DBD), a coiled-coil domain (CDD), and a conserved amino-terminal domain (NTD) (Aigner et al., 2018; Sgrignani et al., 2018).

Cytokines (IL-6, IL-10, and IL-11) or growth factors (EGF, FGF, PDGF, and VEGF) bind to their corresponding cell surface receptors (Iwamaru et al., 2007). These bound receptors form a dimer complex, leading to the initiation and dimerization of glycoprotein 130 (gp130). A complex of the receptors and gp130 recruits Janus kinases (JAKs) and hereby activates JAK/STAT signaling via the phosphorylation cascade. The cytoplasmic phosphorylated tyrosine residues of these receptors create a dock for the STAT3 SH2 domain (Furtek et al., 2016). STAT3 is activated through phosphorylation of Tyr705 located in the SH2 domain. Once activated, pSTAT3 monomers interact via their SH2 domain to form a homodimer of pSTAT3 that dissociates from cytoplasmic partners, translocates to the nucleus, and induces gene transcription (Furtek et al., 2016). In addition to the receptor-associated pathways such as JAKs, phosphorylation of STAT3 can also be triggered by the non–receptor-associated tyrosine kinases (such as Src) (Iwamaru et al., 2007; Sgrignani et al., 2015; Furtek et al., 2016). DNA binding and transcriptional activity of STAT3 depend on the phosphorylation of Ser727 within the STAT3 TAD domain (Siveen et al., 2014).

The function of STAT3 relies significantly on its SH2 domain, which promotes STAT3 homo- or hetero-dimerization, protein–protein interactions, and nuclear translocation of the STAT3 dimers needed for transcription. Thus, the STAT3 SH2 domain mediates the phosphorylation and dimerization of STAT3 due to its association between STAT3 monomers and phospho-tyrosine motifs within relevant receptors (Heppler and Frank, 2019). Due to this important role, the STAT3 SH2 domain becomes a dominating therapeutic target for small molecule modulator discovery and development (Jing and Tweardy, 2005; Wang et al., 2005; Wingelhofer et al., 2018; de Araujo et al., 2019).

(15R,2R)-delavatine A (named as compound 323–1 or 323–1), a natural product with novel cyclopenta*[de]*isoquinoline skeleton, was first reported from the medicinal plant *Incarvillea delavayi*, with subsequently completed total synthesis of (15R,2R)-delavatine A and its chiral isomer (15S, 2R)-delavatine A (named as compound 323–1 or 323–2) (Zhang et al., 2017). Herein, we reported that these compounds modulate the IL-6/STAT3 pathway by 1) inhibition of STAT3 phosphorylation on Tyr705; 2) disruption of STAT3 dimerization by directly targeting its SH2 domain; and 3) inhibition of STAT3 transcriptional activity. Thus, the compounds 323–1 and 323–2 are promising new lead compounds for therapeutic STAT3 inhibition.

## Materials and Methods

### Cell Culture and Reagents

Human prostate cancer cell lines LNCaP and 22Rv1 were purchased from the American Type Culture Collection (ATCC, Virginia, United States) and cultured in RPMI 1640 medium with 10% FCS. HEK 293T and DU145 cells (ATCC) were cultured in a DMEM with 10% FCS. EPT3M1-STAT3 (Qu et al., 2013) were cultured in Ham’s F-12 medium with 10% FCS. Cryptotanshinone and IL-6 were purchased from (Sigma-Aldrich, St. Louis, MI, United States). S3I-201 was bought from Thermo Fisher Scientific (Waltham, MA, United States).

### AlamarBlue Assay for Cell Viability

LNCaP, 22Rv1, and DU145 cells were seeded in 96-well plates for 24 h, followed by treatment with different doses of drugs for 4 days. The alamarBlue assay was performed by adding 10 µL/well alamarBlue cell viability reagent (Thermo Fisher Scientific, MA, United States, cat no. DAL1025) for 4 h, and the absorbance at 570 nm was thereafter recorded using 600 nm as a reference wavelength by using the BioTek Synergy H1 plate reader (Biospx, LA Abcoude, Netherlands). The absorbance data of each group were normalized to the data of the DMSO-treated group to get the relative cell viability. Data were analyzed by Prism software-log (inhibitor) vs. response-variable slope (four parameters) with the formula Y = bottom + (top-bottom)/(1 + 10^((LogIC50-X) *HillSlope)), X: log of dose or concentration, Y: response, decreasing as X increases, top and bottom: plateaus in same units as Y.

### Plasmids and Transfection

HEK 293T cells were transiently transfected with the Cignal STAT3 reporter (SABiosciences, QIAGEN, Venlo, Netherlands) using lipofectamine 3000 transfection reagent (Thermo Fisher Scientific, Waltham, MA, United States) for 24 h, following treatment with 20 ng/ml IL-6 and the indicated concentrations of 323–1, 323–2, S3I-201, or cryptotanshinone. Luciferase activity was measured by using the Dual-Luciferase assay kit (Promega, Madison, WI, United States) using a luminescence microplate reader (BioTek Synergy H1, LA Abcoude, Netherlands). Values were normalized to Renilla luciferase activity of the DMSO vehicle.

### Flow Cytometric Analyses of Cell Apoptosis Assay

DU145 cells were seeded in 6-well plates for 24 h, followed by treatment with various drugs for 72 h. Apoptosis assay was conducted by using the CellEvent™ Caspase-3/7 Green Flow Cytometry Assay Kit (Thermo Fisher Scientific, MA, United States, cat. no. C10427). After removing the media from the cells, 1 µL of CellEvent™ Caspase-3/7 Green Detection Reagent was added to 1 ml of each cell sample to make a final concentration of 500 nM and incubated at RT for 1 h. At the final 5 min of staining, 1 μL of the 1 mM SYTOX™ AADvanced™ dead cell stain solution was added to the samples to a final concentration of 1 μM. The samples were analyzed by using the BD LSRFortessa™ cell analyzer (Franklin Lakes, New Jersey, US).

### Drug Affinity Responsive Target Stability Assay

EPT3M1-STAT3 cells were lysed with cold M-PER buffer (Thermo Fisher Scientific, MA, United States, Pierce cat. no. 78501) containing protease (Roche, Basel, Switzerland, cat. no. 11836153001) and phosphatase inhibitors (Thermo Fisher Scientific, MA, United States, Pierce cat. no. 78420) and centrifuged (18,000 × *g* for 10 min at 4°C). Lysates were diluted to the same final volume and proteolyzed in TNC buffer [50 mM Tris-HCl (pH 8.0), 50 mM NaCl, and 10 mM CaCl2]. Then, 200 µM 323–1 or 200 µM 323–2 or the same volume of DMSO was added and incubated for 1 h at room temperature (RT). A measure of 1.25 mg/ml of pronase solution was diluted serially using 1x TNC buffer to generate 1:300, 1:1,000, and 1:3,000 pronase stock aliquots. Pronase was added into both the DMSO and drug groups and incubated for 30 min at RT. Digestion was stopped by adding 4X loading buffer and heating to 90°C for 10 min immediately prior to the Western blot assay, according to publications (Lomenick et al., 2009; Lomenick et al., 2011).

### Indirect Immunofluorescence Staining

EPT3M1-STAT3 cells (grown on coverslips) were treated with DMSO, 20 µM 323–1 or 323–2, 100 µM S3I-201, or 5 µM cryptotanshinone for 24 h. Cells were fixed with 4% paraformaldehyde, washed with PBS, and permeabilized with cold methanol. Primary and secondary antibodies were described, cat. no. and dilutions (blocking, PBS washes, or reference to your previous publication (or Supplementary Materials)), and mounted onto millipore microscope slides with 7 μL of ProLong^®^ Gold Antifade Mountant with DAPI (Thermo Fisher Scientific, MA, United States, **cat no.** P36935). Images were captured using the Leica DMRBE microscope or Cytation5 Cell Imaging Multi-Mode Reader.

### Deoxyribonucleic Acid Binding Assay

DU145 cells were seeded and treated with 5–20 µM 323–1 or 323–2 or 100 µM S3I-201 for 24 h. Nuclear extraction was performed according to the STAT Family Transcription Factor Assay Kit (Abcam, Cambridge, United Kingdom, cat. no. ab207228) (Supplementary Materials).

### Western Blot Analysis

The levels of expressions of phosphorylated STAT3 (pSTAT3; Y705), pSTAT1 (pSTAT1; Y701), and pSTAT1 (S727) proteins were determined by Western blotting, following the procedures described (Liu L. et al., 2014). The following antibodies from Abcam, Cambridge, United Kingdom, and dilutions used in Western blotting are as follows: anti-pSTAT3 (Tyr705) (ab76315, 1/5,000); anti-total STAT3 (ab119352, 1/2,500); anti-pSTAT1 (Y701) (ab30645, 1/500); anti-pSTAT1 (S727) (ab86132, 1/500); anti-total STAT1 (ab47425, 1/500); anti-JAK2 (ab108596, 1/1,000); anti-SRC (ab47405, 1/500); anti-PARP p85 Fragment (G7341, 1/1,000); anti-MCL1 (ab32087, 1/1,000); anti-cyclin-D1 (ab10540, 1/500); anti-BCL-XL (ab32370, 1/500); anti-survivin (ab76424, 1/2000); anti-β-actin (ab8226, ab8227, 1/2000); anti-GAPDH (ab181602, 1/2,500); anti-HA (ab9110, 1/5,000); and anti-FLAG (DDDDK tag) (ab1162, 1/10,000).

### Co-Immunoprecipitation

For this procedure, 7.5 × 10^6^ HEK 293T cells were transfected with 10 µg/10 cm dish FLAG-STAT3 and 10 µg/10 cm dish HA-STAT3 plasmids for 24 h, followed by treatment with 20 µM 323–1 or 323–2 and 100 µM S3I-201 for 4 or 24 h Thereafter, 293T cells were collected in the medium and centrifuged at 800 g × 5 min and then washed with cold PBS twice before adding 500 μL Pierce™ IP lysis buffer (Thermo Fisher Scientific, MA, United States, cat. no. 87787), containing protease and phosphatase inhibitor cocktail without DTT (Roche, Basal, Switzerland, cat. no. 11836153001) into the cells. Cell lysates were passed several times through a 27^1⁄2^-gauge needle to disrupt the nuclei. Then, 1–2 mg extracts were added with a pre-cleaned 50 µL slurry of Pierce™ DYKDDDDK magnetic agarose (Thermo Fisher Scientific, MA, United States, cat. no. A36797), and then, supernatants were collected with magnet and immunoprecipitated (IP) at RT on a rotator for 30 min. Beads were boiled at 95°C for 5 min followed by the addition of 100 μL of 1x non-reducing sample buffer (Thermo Fisher Scientific, MA, United States, cat. no. 39000) into beads. The supernatants were collected with a magnet and then added 5 μL of 1M DTT (Thermo Fisher Scientific, MA, United States, cat. no. P2325) into samples before proceeding with immunoblotting.

### Computational Docking of STAT3

The computational docking of 323s to three-dimensional crystal structures of STAT3, including the phosphorylated (PDB entry 1BG1) and the non-phosphorylated (PDB entry 3CWG) structure, was utilized by the molecular docking tool Maestro 9.0 Glide, as illustrated previously (Zheng et al., 2018). The low-energy conformers of 323s were generated by the LigPrep module of Maestro, while the protein models were established via removing crystallized solvent molecules, re-assigning the bond order, and supplementing hydrogen atoms. The minimization of energy in an OPLS-2005 force field was applied with an RMSD value less than 18 Å, and the number of conformations was set to 100. The docking simulation applied three pockets of the STAT3 SH2 domain to the binding site. The calculation of the docking score (kcal/mol) was conducted to verify the capacity of protein–ligand interactions.

### Fluorescence Polarization Assay

The FP assay was conducted as described in Zhao et al. (2010). The fluorochrome-labeled phosphopeptide, 5-FLU-G (pY)LPQTV-NH2, was synthesized by ProImmune (London, United Kingdom) with over 95% purity as a probe. The recombinant human STAT3 protein was purchased from Abcam (ab43618). To obtain an inhibitory effect, drugs (0–1,000 μM) and 150 nM human STAT3 were incubated at 37°C in an assay buffer (50 mm NaCl, 10 mm HEPES, 1 mm EDTA, and 0.01% Triton X-100) for 1 h prior to co-incubation with 10 nM of the fluorochrome-labeled peptide at RT for 30 min. The plate was sealed under nitrogen and left at RT for 72 h. The fluorescence polarization was then analyzed by the BioTek Synergy™ Neo2 Multi-Mode Microplate Reader (BioTek Instruments, Winooski, VT, United States). Binding curves were fit to a log-logistic function using the R package *drc.* Ki was determined from the resulting IC50 using the Cheng–Prusoff equation and the Kd for fluorochrome-labeled peptide binding to STAT3, Ki = IC50/(([L])/Kd) +1. The assays were performed at the high-throughput chemical biology screening platform (www.med.uio.no/english/research/core-facilities/chemical-biology-screening/) Centre for Molecular Medicine Norway (NCMM), University of Oslo.

### Antitumor Activity *In Vivo*

Four-week-old male BALB/c-nu nude mice (15–20 g) were obtained from the Shanghai BiKai Laboratory Animal Co., Ltd. (Shanghai, China). The animals were maintained under specific pathogen-free conditions with food and water supplied ad libitum in the Laboratory Animal Center of the Second Military Medical University, Shanghai. All animal experiments were carried out in accordance with the Guide for the Care and Use of Laboratory Animals of the National Institutes of Health and were approved by the Committee on the Ethics of Animal Experiments of the Second Military Medical University, China. LNCaP cells were harvested and resuspended in PBS. A total of 5 × 10^6^ cells were subcutaneously injected into the right flank. Tumor volume was calculated using the following formula: V = (L × W^2^)/2, where L is the length and W is the width of the tumor nodules measured with vernier calipers. Once the volume of the tumors reached 75–100 mm^3^, the mice were randomly divided into three groups (*n* = 6). The mice were treated daily for 3 weeks with IP injections of either vehicle (olive oil) or 323–2 (20 mg/kg or 40 mg/kg in the vehicle). Body weights and tumor volumes were measured before each drug injection. After the 22nd day, the mice were euthanized, and the tumors were isolated, weighed, and photographed.

### Statistics

Significance in groups was determined by using one-way ANOVA multiple comparison. *p ≤ 0.05, **p ≤ 0.01, ***p ≤ 0.001, and ****p ≤ 0.0001.

## Results

### Compounds 323–1 and 323–2 Selectively Modulated the STAT3 Pathway

**FIGURE 1:**
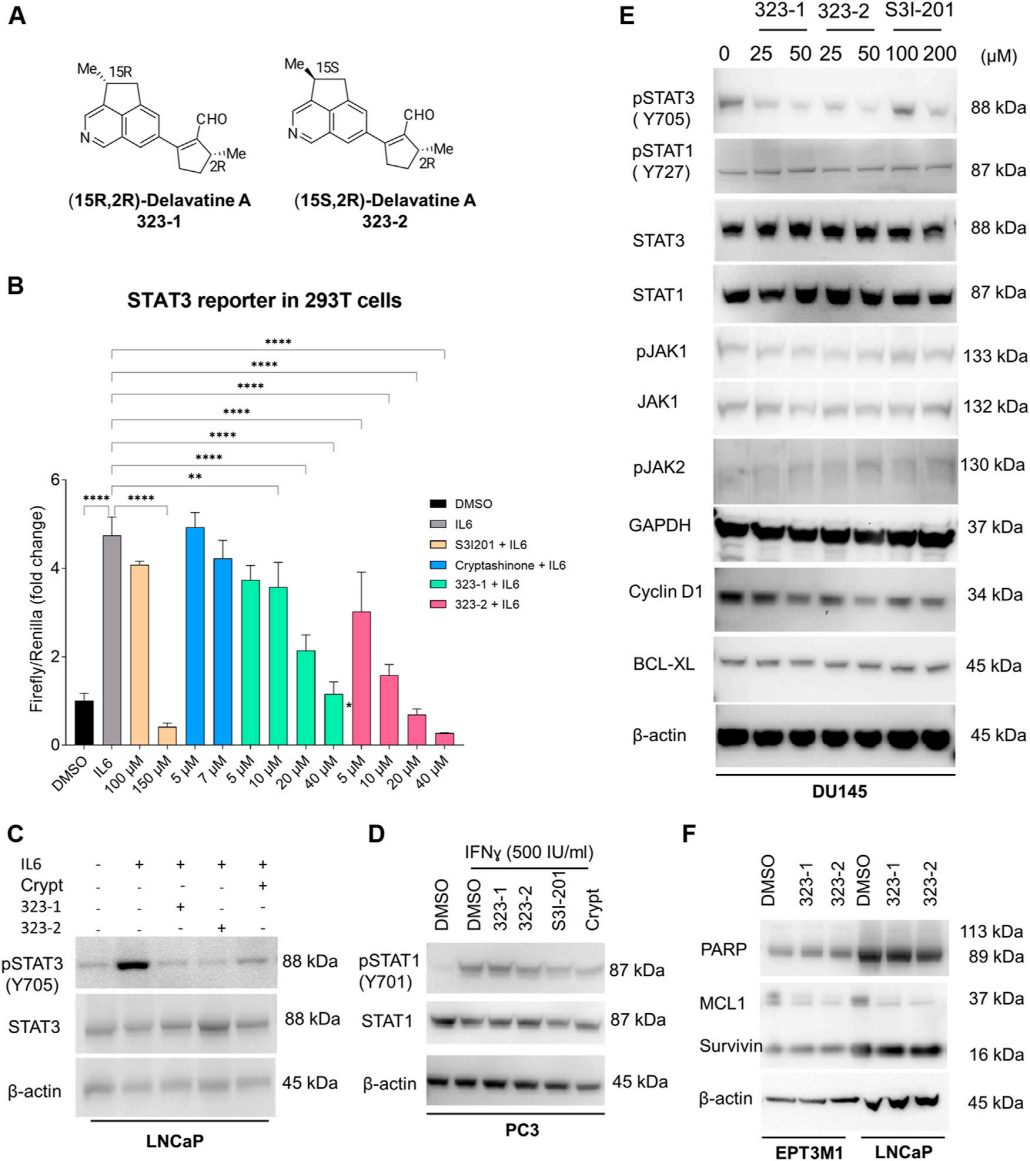
Compounds 323–1 and 323–2 target the IL-6/STAT3 pathway. **(A)** Chemical structure and nomenclature of compounds 323–1 and 323–2. **(B)** HEK 293T cells transfected with the STAT3 luciferase reporter were treated with different doses, as indicated of 323–1 or 323–2, S3I-201, or cryptotanshinone (Crypt) in the presence of 20 ng/ml IL-6 for 24 h. All data are represented as the average ±s. e.m. (*n* = 3).Significance was analyzed by using one-way ANOVA multiple comparison. *p ≤ 0.05, **p ≤ 0.01, ***p ≤ 0.001, and ****p ≤ 0.0001. **(C)** LNCaP cells were treated with DMSO, 20 µM 323–1 or 323–2, or 5 µM cryptotanshinone for 24 h before treatment with or without 10 ng/ml IL-6 for the last 15 min. **(D)** PC3 cells were treated with DMSO, 20 µM 323–1, 20 µM 323–2, 100 µM S3I-201, or 5 µM cryptotanshinone for 24 h before treatment with or without 500 IU/ml IFNɣ for the last 15 min. **(E)** DU145 cells were treated with indicated doses of 323–1, 323–2, or S3I-201 for 24 h. **(F)** EPT3M1-STAT3 or LNCaP cells were treated with vehicle (DMSO), 20 µM 323–1, and 20 µM 323–2 for 48 h, respectively. **(C–F)** Lysates were analyzed by Western blotting and indicated antibodies. Data are shown as the representative results of three separate repeats.

In a screen of nearly 600 natural compounds, (15R,2R)-delavatine A (compound 323–1) and (15S,2R)-delavatine A (compound 323–2) (Figure 1A) were found to efficiently inhibit the proliferation of tumor cells. The total synthesis of 323s was conducted as previously reported (Zhang et al., 2017). The chemical structures of 323s were validated by ^1^H NMR, ^13^C NMR, and chiral HPLC chromatography (see Supplementary Tables S1 and S2 and Supplementary Figures S1–S6) with optical rotations +62.0 (*c* = 0.05 in CHCl_3_) and -6.7 (*c* = 0.05 in CHCl3), respectively (in the Supplementary Material). As shown in Supplementary Figure S7A, different prostate cancer cell lines were inhibited in a dose-dependent manner after treatment with 323s for 96 h. Compared with the commercial STAT3 inhibitor cryptotanshinone, 323s showed weaker cytotoxicity with a less IC50 value in all three cell lines. Meanwhile, drug resistance was observed for another commercial STAT3 inhibitor S3I201 in DU145 cells with an IC50 value of 1,014 μM and a similar effect as 323s to inhibit tumor cell proliferation in LNCaP cells. These data indicated that 323s possessed less cytotoxicity.

**FIGURE 2:**
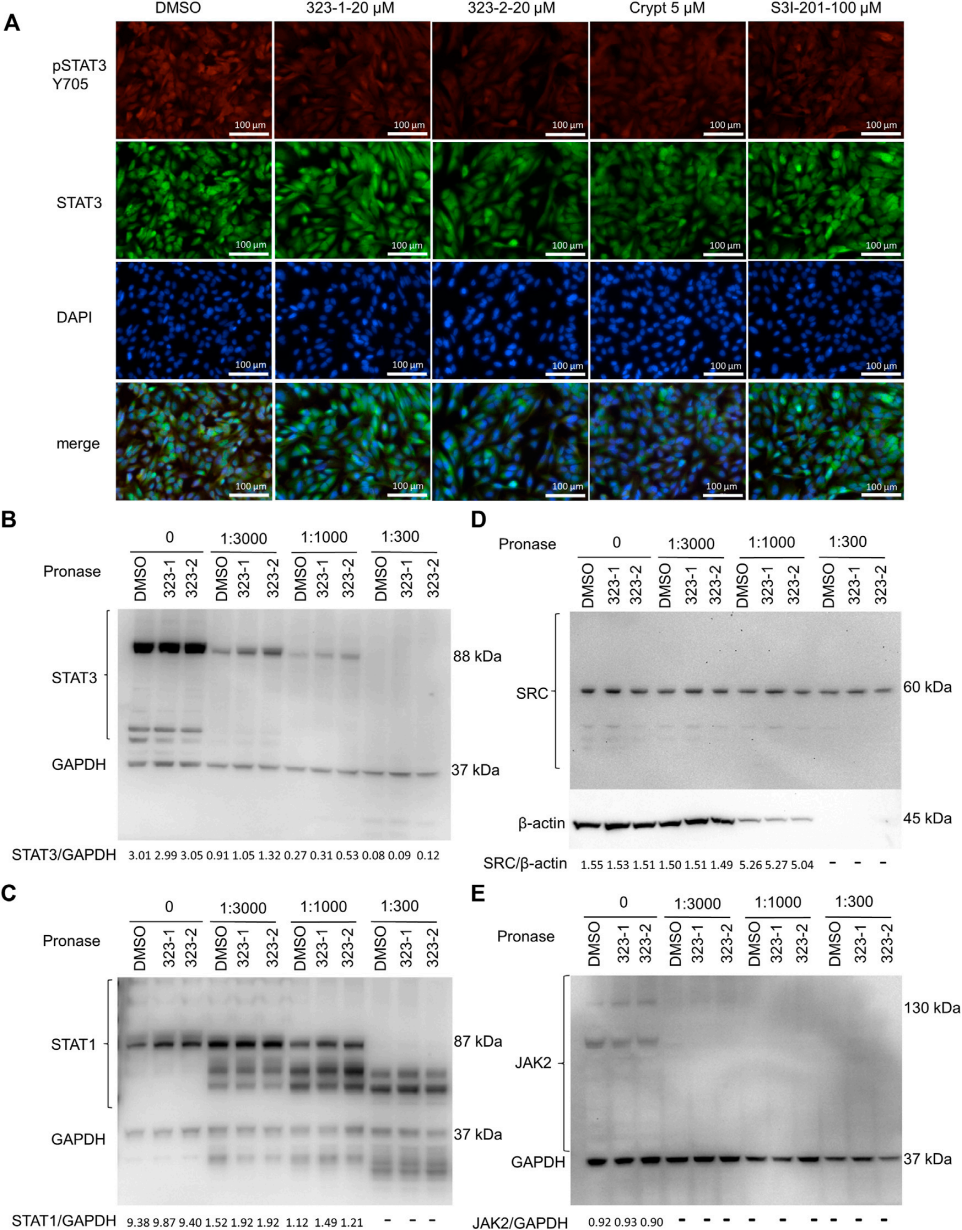
Compounds 323–1 and 323–2 target pSTAT3 over pSTAT1. **(A)** Indirect immunofluorescence microscopy of EPT3M1-STAT3 cells. Cells were treated with DMSO, 20 µM 323–1, 20 µM 323–2, 5 µM cryptotanshinone (Crypt), or 100 µM S3I-201 for 24 h. They were incubated with the primary antibody anti-GFP (ab290) and secondary antibody goat anti-rabbit IgG (H + L) (SouthernBiotech 4,050–02), respectively. Representative results of three independent repeats are shown. **(B–E)** DARTS assay: 323–1 and 323–2 directly target STAT3. EPT3M1-STAT3 cell lysates were prepared as described in (Lomenick et al., 2009) and incubated with 200 μM 323–1 or 200 μM 323–1 or an equivalent amount of vehicle (DMSO) for 1 h, followed by digestion with 1.25 mg/ml pronase at dilution ratios of 1:3,000, 1:1,000, and 1:300 for 1 h by using 1x TNC buffer to create serial pronase stock aliquots. Samples were subjected to Western blotting with antibodies against **(B)** STAT3 (ab119352); **(C)** STAT1 (ab47425); **(D)** SRC (ab47405); **(E)** JAK2 (ab108596) or **(B)**, **(C)**, or **(E)** GAPDH (ab181602). Data are shown as representative results of three experiments.

Herein, we sought to determine whether 323–1 and 323–2 were able to suppress STAT3 phosphorylation and activation. First, to confirm the inhibitory effect of 323s on STAT3 activity, luciferase assays were carried out using a STAT3-luciferase reporter vector in 293T cells, following treatment with 323–1, 323–2 or the commercial STAT3 inhibitors S3I-201 and cryptotanshinone in the presence of IL-6 for 24 h (Figure 1B). 323–1 and 323–2 exhibited more potent inhibition of STAT3 transcriptional activity than cryptotanshinone and S3I-201 (Figure 1B). The expression of STAT3 and STAT1 was tested in different cell lines, and three cell lines (LNCaP, DU145, and EPT3M1-STAT3) expressed high basal levels of STAT3 whereas 6 cell lines (293T, 22Rv1, LNCaP, PC3, DU145, and EPT3M1-STAT3) showed a basal STAT1 expression (Supplementary Figure S7B, C). Among these cell lines, IL-6-treatment for 15 min induced phosphorylation of STAT3 on Tyr705 only in LNCaP cells but not in 22Rv1, DU145, or EPT3M1-STAT3 cells, without affecting total STAT3 (Supplementary Figure S7D). Figure 1C shows that 323–1 and 323–2 inhibited IL-6-induced phosphorylation of STAT3 on Tyr705 in LNCaP cells but not phosphorylation of STAT1 on Tyr701 induced by IFN-ɣ in PC3 cells (Figure 1D). Notably, 323–1 and 323–2 demonstrated a selective inhibitory effect of phosphorylated STAT3 (Tyr705) over pSTAT1 (S727) and impaired the expression of phosphorylated STAT3 at Tyr705 with a better effect than S3I-201 in DU145 cells (Figure 1E). The upstream protein JAKs, such as pJAK1, pJAK2, and JAK1, were not affected by both compounds (Figure 1E). Results shown in Figure 2A further confirmed inhibitory effects on STAT3 (Tyr705) phosphorylation by indirect immunofluorescence assay using the phospho-STAT3 (Tyr705) primary antibody and direct GFP detection of EPT3-M1 cells with a high expression of fusion GFP-STAT3. Taken together, these findings suggest that 323–1 and 323–2 are small-molecule inhibitors of STAT3.

### Compounds 323–1 and 323–2 Reduced the Protein Level of STAT3 Target Genes MCL1 and Cyclin D1

Aberrant STAT3 regulates the expression of downstream target genes involved in anti-apoptosis (Bcl-2, Mcl-1, survivin, and Bcl-xL), cell cycle (cyclin D1 and c-Myc), angiogenesis (VEGF and HIF1α), invasion and metastasis (MMP-1, MMP-2, and MMP-9), and the inhibition of host immune surveillance (Siveen et al., 2014; Furtek et al., 2016; Beebe et al., 2018; Su et al., 2018; Owen et al., 2019). To further identify the properties of 323–1 and 323–2, we treated DU145, EPT3M1-STAT3, and LNCaP cells with compounds 323–1 and 323–2, followed by Western blotting. Figure 1E shows that the expressions of cyclin D1, but not BCL-XL, in LNCaP cells were downregulated dose-dependently by these two compounds. Compounds 323–1 and 323–2 repressed the protein level of the STAT3 target gene MCL1 in both cell lines, but not survivin or PARP, in EPT3M1-STAT3 and LNCaP cells (Figure 1F). The results suggested that compounds 323–1 and 323–2 modulate STAT3 target gene expression.

### Compounds 323–1 and 323–2 Did Not Affect the Binding of STAT3 to Deoxyribonucleic Acid

Several available STAT3 inhibitors target the DBD or SH2 domains of STAT3 or inhibit activating phosphorylation of Tyr705. Due to the highly conserved region of the SH2 domain, such as in Src, compared with other proteins and the insufficient capability of the available STAT3 SH2 domain inhibitors (Son et al., 2017; Huang et al., 2018), the interest in targeting STAT3 DBD is growing. To determine if the compound 323 targets the STAT3 DBD, nuclear lysates were extracted from the cells treated with drugs for 24 h and incubated with oligonucleotides containing STAT consensus binding sites, followed by the relevant primary (STAT1, STAT3, STAT5a, or STAT5b) antibodies. Different from the STAT3 DBD inhibitor S3I-201, neither 323–1 nor 323–2 affected the DNA-binding domain of STAT1, STAT3, STAT5A, and STAT5B (Supplementary Figure S8A).

### Compounds 323–1 and 323–2 Targeted STAT3 Directly and Disrupted STAT3 Dimerization

To directly address if compounds 323–1 and 323–2 target STAT3, the DARTS assay was used, based on the observation that proteins bound by drugs may be more stable against protease degradation. In Figures 2B–E, Western blotting showed protection of the target protein STAT3, whereas digestions of the non-target proteins STAT1, JAK2, SRC, and GAPDH were unchanged by incubation with 323–1 and 323–2. This suggests that 323–1 and 323–2 bind to STAT3 directly.

**FIGURE 3:**
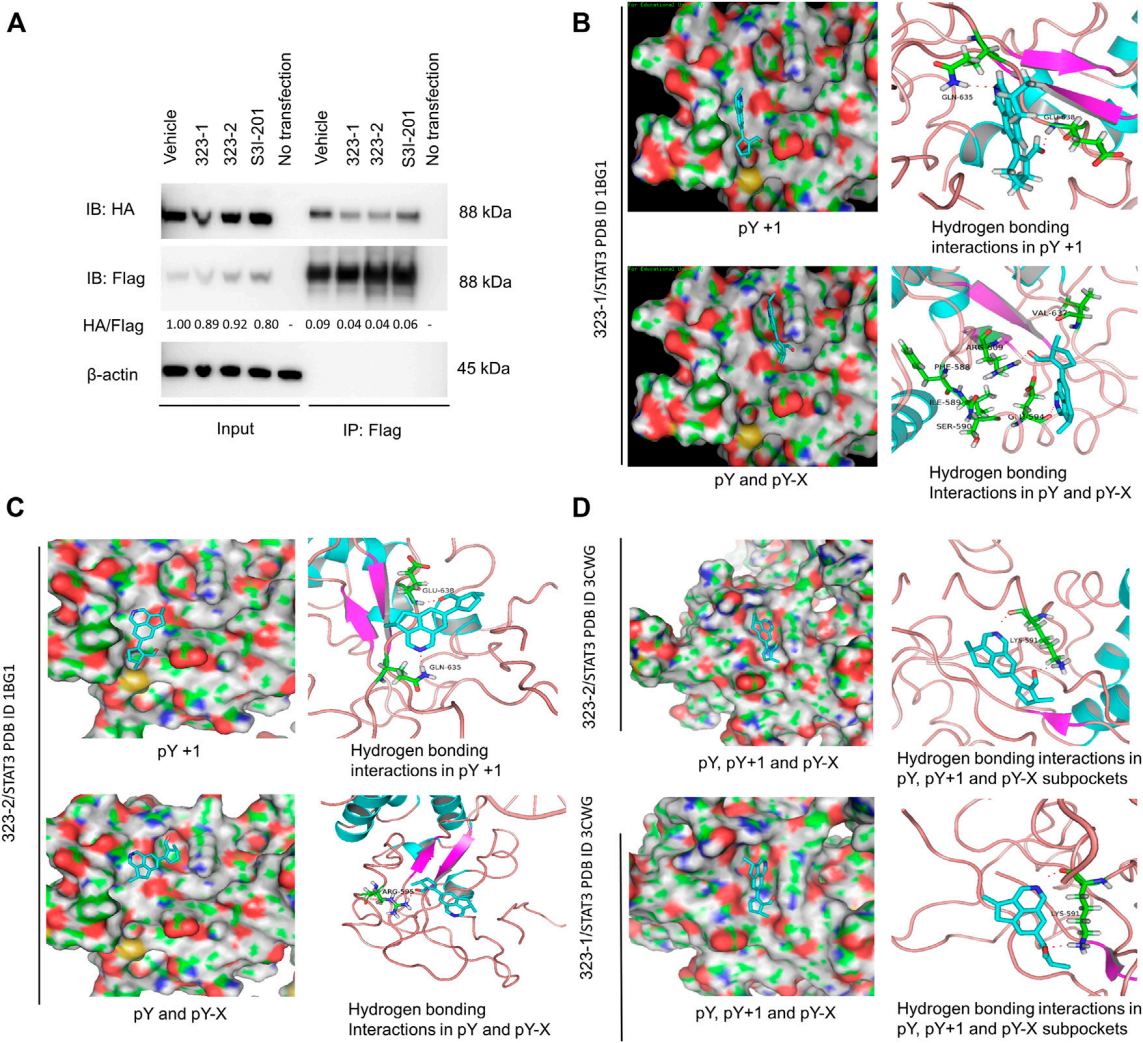
Compounds 323–1 and 323–2 block STAT3 dimerization and target the STAT3 SH2 domain. **(A)** HEK 293T cells were transfected with FLAG-STAT3 and HA-STAT3 and were treated with vehicle (DMSO), 50 µM 323–1, 50 µM 323–2, and 200 µM S3I-201 for 24 h. Extracts were immunoprecipitated with the anti-FLAG antibody, followed by immunoblotting with anti-HA, anti-FLAG, and anti-β-actin (ab8226). The HA/FLAG ratio was calculated by ImageJ software, and the vehicle within the input group was set as control. Data are shown as representative results of three experiments. **(B, C)** *In silico* computational docking analysis by Maestro 9.0 Glide software selected 323–1 **(B)** or 323–2 **(C)** as ligands and STAT3 protein (PDB: 1BG1). The key amino acid residues are used in the positive control as the center of the grid. The center of the grid is less than 18 Å, docking Select XP for precision, and selected 100 output constellations. **(D)** Molecular docking of compounds 323–1 and 323–2 on STAT3 (PDB: 3CWG) is previously illustrated.

Nuclear translocation of the STAT3 dimers mediates STAT3 transcriptional activity. In order to test whether compound 323s are STAT3 dimerization inhibitors, 293T cells were transiently transfected with HA-tagged STAT3 and FLAG-tagged STAT3 expression plasmids, then stimulated with 323s or S3I-201 for 24 h, and next immunoprecipitated with the anti-FLAG antibody to pull down the STAT3-associated proteins, which were finally subjected to Western blot with anti-HA, anti-FLAG, or anti-β-actin antibodies. As exemplified in Figure 3A, it was confirmed that 323s could disrupt the dimerization of STAT3 compared with S3I-201.

### Molecular Docking Data Supported that 323–1 and 323–2 Target the SH2 Domain of STAT3

The STAT3 SH2 domain (residues 583–688) contains three sub-pockets: PY, PY+1, and PY + X. The STAT3 SH2 domain facilitates the phosphorylation and dimerization of STAT3 due to its association between STAT3 monomers and phospho-tyrosine motifs within relevant receptors (Heppler and Frank, 2019). To date, the STAT3 SH2 domain has become a dominating therapeutic target for small-molecule modulator discovery and development (Jing and Tweardy, 2005; Wang et al., 2005; Wingelhofer et al., 2018; Bai et al., 2019; da Silva Junior et al., 2019; de Araujo et al., 2019; Igelmann et al., 2019). Several new STAT3 SH2 domain binding inhibitors have been identified through *in silico* computational screening assay by docking compounds into the STAT3 SH2 domain, such as S3I-201 (Siddiquee et al., 2007; Zhang et al., 2010; Zhang et al., 2013; Liu LJ. et al., 2014).

To confirm that 323s bind to STAT3, we used *in silico* computational modeling and the fluorescence polarization (FP) assay. Computational docking and molecular dynamics simulation identified potential binding sites for 323–1 and 323–2 in the STAT3 SH2D (Figures 3B–D). In the molecular docking with phosphorylated (PDB entry 1BG1) STAT3, both compounds appeared to have potential binding ability to three pockets of the STAT3 SH2 domain, PY, PY+1, and PY-X, whereas S3I-201 appeared to be able to bind only to the PY and PY-X pockets, not the PY+1 pocket. In Figure 3B, the nitrogen of the isoquinoline group in 323–1 forms a hydrogen bond with Gln635, and the carbonyl oxygen on the formaldehyde group forms a hydrogen bond with Glu638. The pY+1 pocket is mainly a hydrophobic region, and the small-molecule skeleton cyclopenta [*de*] isoquinoline is facing this pocket, forming a hydrophobic interaction with surrounding amino acid residues (IIe634, Ser636, and Val637). The nitrogen of the isoquinoline group in 323–1 forms a hydrogen bond with Glu594, the carbonyl oxygen on the formaldehyde group faces the pY binding pocket and forms a hydrogen bond with Arg609, and Arg609 can form a direct polarity with phosphotyrosine 705. Arg609 can inhibit the binding of SH2 to phosphotyrosine 705. The small-molecule skeleton cyclopenta [*de*] isoquinoline faces the pY-X binding pocket and interacts hydrophobically with adjacent amino acid residues (Phe588, IIe589, Ser590, and Val637). Similarly, compound 323–2 forms a bond with PY, PY+1, and PY-X pockets (Gln635, Glu638, IIe634, Ser636, Val637, Arg595, and Lys591) in the STAT3 SH2 domain (Figure 3C).

It is reported that non-phosphorylated STAT3 may also form dimers, enter the nucleus from the cytoplasm to bind to DNA, and activate transcription (Timofeeva et al., 2012; Butturini et al., 2016). In docking with the non-phosphorylated (PDB entry 3CWG) STAT3 structure, both 323–1 and 323–2 were predicted to directly interact with the Tyr(P)-binding subpocket via hydrogen bonding within residues Lys591 (Figure 3D). Also, the cyclopenta [*de*] isoquinoline of 323s associated with the pY-X binding pocket bound by tetrahydro cyclopentane and formed hydrophobic interactions lined by residues Phe588, IIe589, Ser590, and Val637. These data suggest that 323s are STAT3 SH2 domain inhibitors by disrupting STAT3 dimerization and further supporting the results of the DARTS assay.

### Compounds 323–1 and 323–2 Displaced the Binding of STAT3 to Fluorescein-Labeled Phospho-Tyr-Peptide GpYLPQTV-NH2

The *in silico* predictions were further examined by competition binding FP assays, which detected the displacement by drugs of the binding of labeled phosphopeptide, 5-FLU-GpYLPQTV-NH2, to the human recombinant STAT3 protein. The phosphotyrosine peptide GpYLPQTV corresponds to the residues 903–909 within the gp130 subunit of the IL-6 receptor, which has been validated to bind the STAT3-SH2 domain according to the method (Haan et al., 1999; Ren et al., 2003; Zhang et al., 2013). Strategies to target STAT3 SH2 have been pursued recently by various groups. These small-molecule inhibitors are designed to bind to a site resident in the STAT3 SH2 domain by competing with the pY705 (Jing and Tweardy, 2005).

**FIGURE 4:**
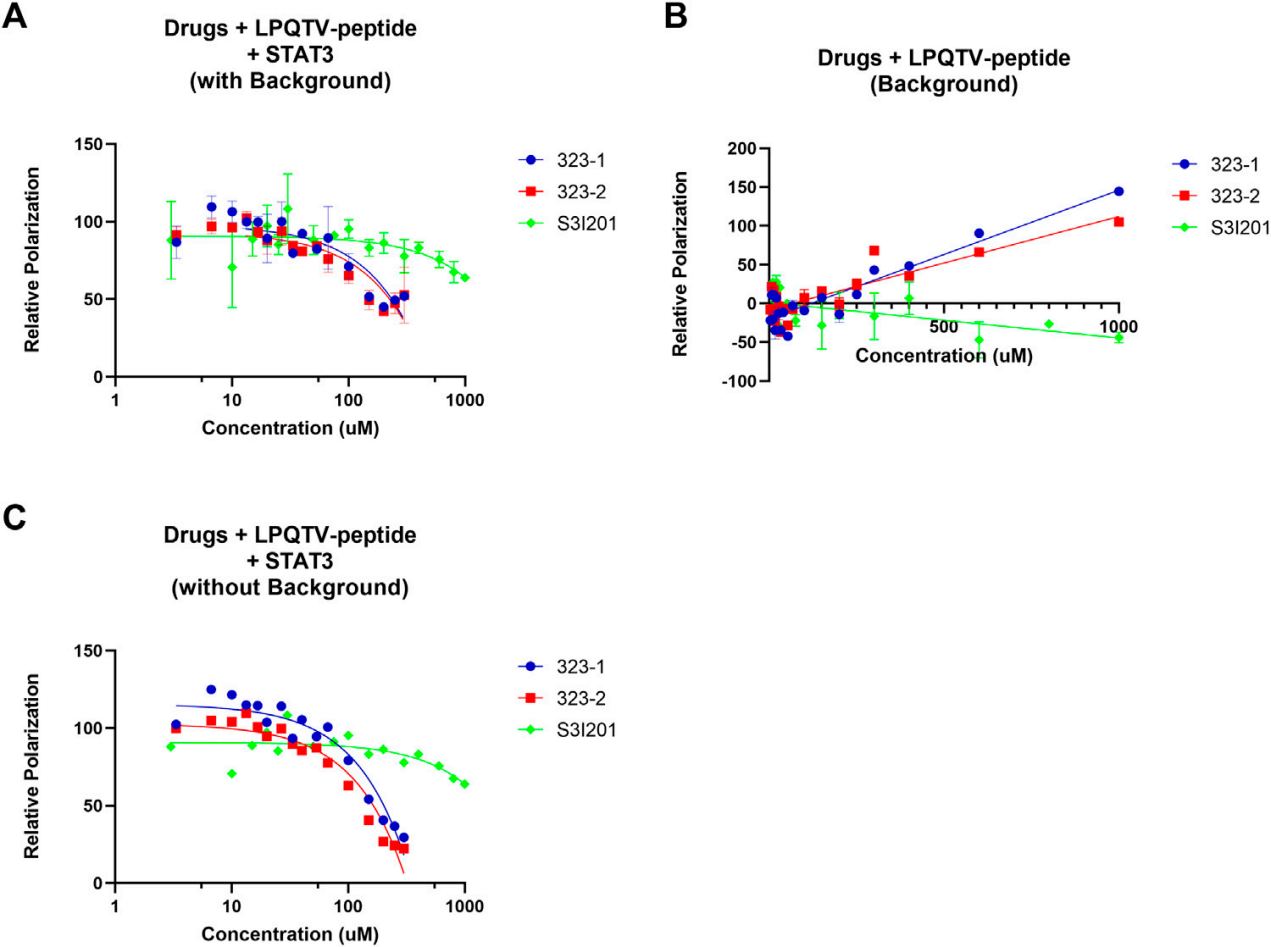
Fluorescence polarization (FP) assays identified 323s as direct STAT3 SH2 domain binders. **(A)** Fluorescence polarization (FP) analysis of the binding of 10 nM GpYLTQTV-NH_2_and 150 nM human STAT3 protein with increasing concentration of drugs. **(B)** Different doses of drugs combined with only 10 nM GpYLTQTV-NH_2_ **(B)** or 150 nM human STAT3 protein. **(C)** FP analysis of background deduction by application of 10 nM GpYLTQTV-NH2 and increasing doses of drugs. Control was set as 1% DMSO. Data are representative of two independent experiments.

As STAT3 may be recruited to gp130 within phosphotyrosine residues (Stahl et al., 1995), we utilized the competitive FP assay to identify whether 323–1 and 323–2 target the STAT3 SH2 domain. Figure 4A shows that 323–1 and 323–2 disrupted the binding of STAT3 to the phosphotyrosine peptide GpYLPQTV with Ki values of around 94 and 75 μM, respectively. The potency of 323s to disrupt the STAT3/GpYLPQTV was around five-fold higher than the S3I-201 Ki values of around 529 μM (Zhao et al., 2010). Notably, both 323–1 and 323–2 exhibited an increase in polarization at concentrations above 200 µM. These compounds do not appear to be fluorescent at the measured wavelengths, so we hypothesized that some interaction between drug and fluorophore that decreases the fluorophore’s mobility occurs at high concentrations. This was checked by measuring the polarization of the peptide in the presence of high concentrations of the drug in the absence of STAT3 (Figure 4B). These polarization responses were then subtracted as background from the binding curves of ligands to STAT3 in the presence of reporter peptide. The background-subtracted curves were then used to validate the efficiency of protein–ligand interaction with Ki values of 94 µM for 323–1 and 75 µM for 323–2 (Figure 4C).

Taken together, these data confirm that 323–1 and 323–2 are direct STAT3 SH2 inhibitors by disrupting the binding of the STAT3 phosphotyrosine-peptide, corresponding with the computational docking and DARTS assay.

### Compounds 323–1 and 323–2 Inhibited Colony Formation and Induced Apoptosis of Prostate Cancer Cells

**FIGURE 5:**
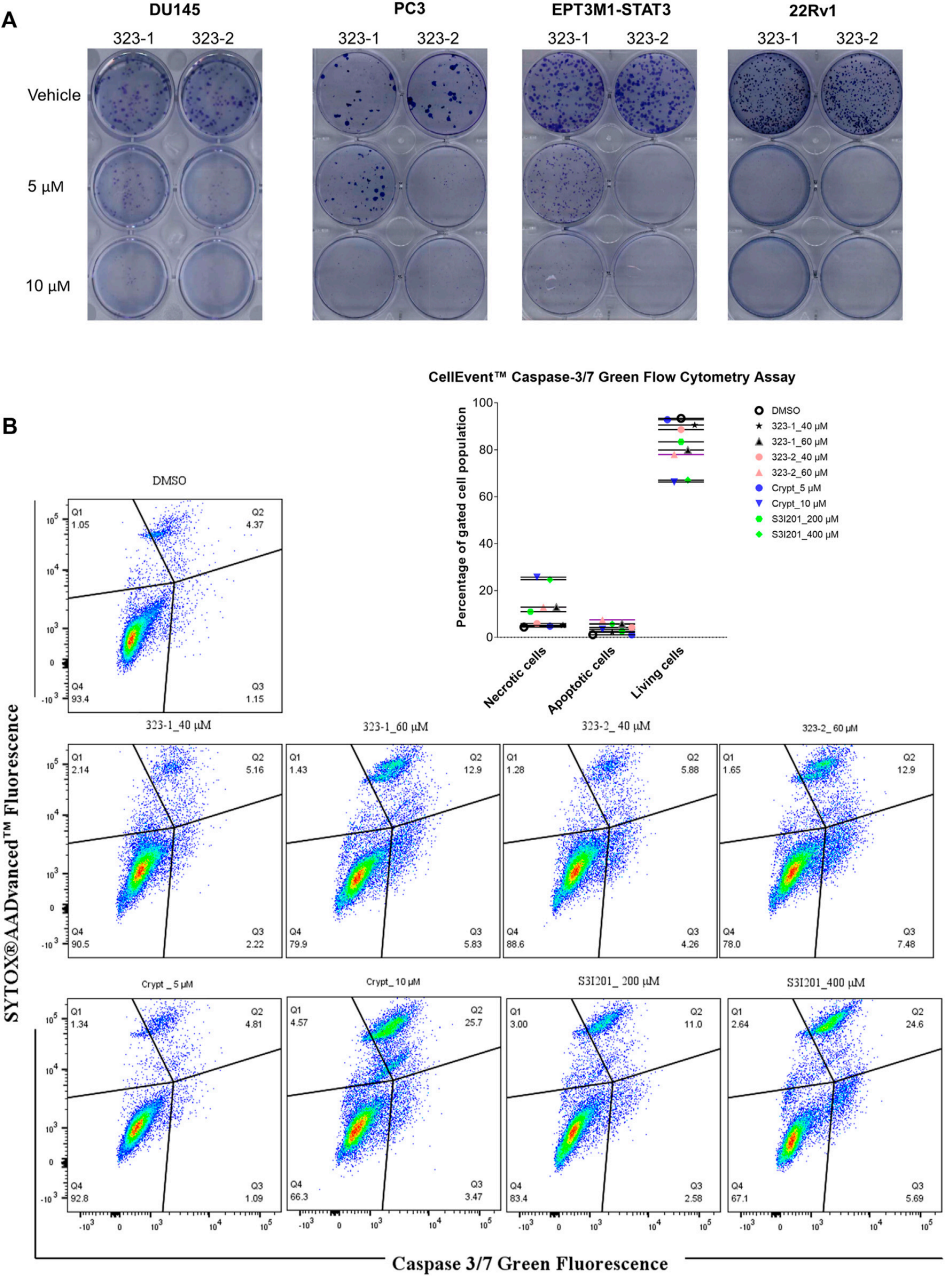
Compound 323s reduced the clonogenicity and induced apoptosis. **(A)** On the 2nd day after seeding, compounds 323–1 and 323–2 were added and DU145, PC3, EPT3M1-STAT3, and 22Rv1 cells were allowed to grow for 2 weeks to form colonies and next stained with 0.4% crystal violet (w/v). **(B)** Flow cytometric analyses of cell apoptosis induced by treatment with vehicle (DMSO), 323–1, 323–2, cryptotanshinone, or S3I201 at indicated concentrations for 72 h in DU145 cells. Data are representative results of three experiments.

The effect of compounds 323–1 and 323–2 concerning the inhibition of clonogenicity was investigated *via in vitro* clonogenic assays, which correspond to tumorigenicity in nude mice (Freedman and Shin, 1975). On the second day after seeding single cells, 323–1 and 323–2 were added at various concentrations, and cells were allowed to grow for 2 weeks to form colonies and then stained with 0.4% crystal violet (w/v). Concentration-dependent inhibition of colony formation in four cancer lines, DU145, PC3, EPT3M1-STAT3, and 22Rv1 cells, was observed after exposure to compounds 323–1 and 323–2 in Figure 5A. The ability of cells to retain their reproductive integrity was effectively blocked by 323s.

One of the effects of antitumor drugs is to induce apoptosis in cancer cells. We next evaluated the anti-apoptotic effect of 323s in DU145 cells. Caspases 3 and 7 are regarded as vital modulators of mitochondrial events of apoptosis. As shown in Figure 5B, compounds 323–1 and 323–2 induced apoptosis-like changes in many cells and appeared to induce apoptosis in DU145 cells treated for 72 h. By contrast, induction of necrosis by 323s at 40 µM was much weaker than by the working concentration of cryptotanshinone (10 µM), which is reported to possess a high cytotoxic effect. Meanwhile, 200 µM of S3I201 significantly enhanced the percentage of necrotic cells compared with 40 µM 323s. These results indicate that 323s possess *in vitro* anti-tumor activity by inhibition of cell proliferation, clonogenicity, and apoptosis induction.

### Compound 323s Impeded Tumor Growth in the Mouse Model

**FIGURE 6:**
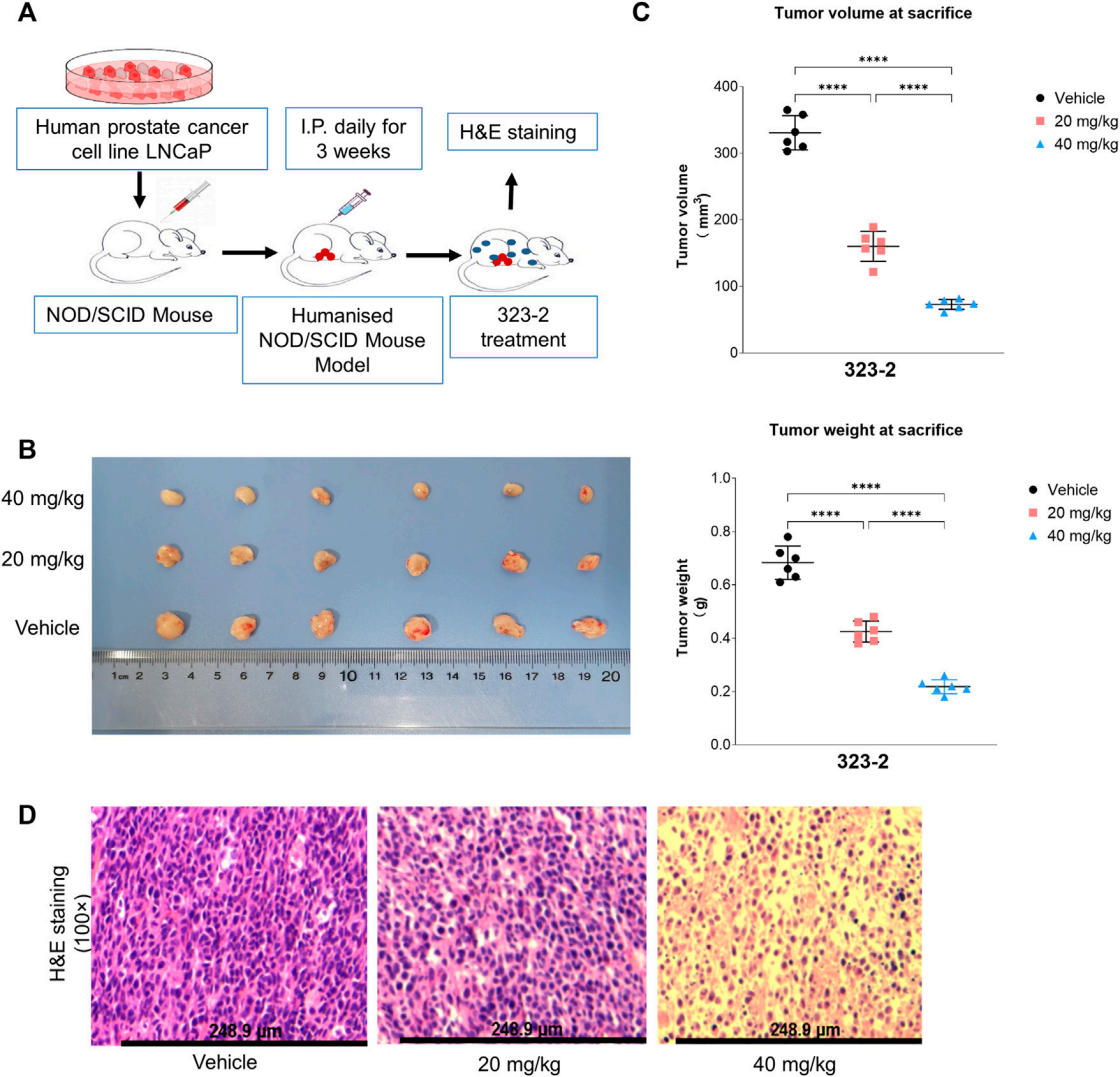
Compound 323s repressed growth of LNCaP xenografts. **(A)** Workflow of the *in vivo* mouse tumor experiment. LNCaP cells were harvested and resuspended in PBS. A total of 5 × 10^6^ cells were subcutaneously injected into the right flank of BALB/c-nu nude mice. The mice were treated daily for 3 weeks with IP injections of either the vehicle (olive oil) or the compound 323–2 (20 mg/kg or 40 mg/kg in the vehicle). Body weights and tumor volumes were measured before each drug injection. After the 22nd day, the mice were sacrificed, and the tumors were collected, weighed, and stored at −70°C. **(B)** Photograph of the harvested LNCaP tumors from each group on the day of sacrifice. **(C)** Individual animal tumor volume on the day of sacrifice in each group. **(D)** H&E staining of harvested representative LNCaP tumors in each group. Significance was determined by using one-way ANOVA multiple comparison. *p ≤ 0.05, **p ≤ 0.01, ***p ≤ 0.001, and ****p ≤ 0.0001.

As shown in Figure 6A, a human prostate cancer cell line LNCaP containing the full-length AR gene was injected into NOD/SCID mice to establish an *in vivo* tumor model. Compound 323s were administrated i.p. daily to validate the effect on tumor growth. The *in vivo* data supported that 323s significantly inhibited tumor growth by measuring the tumor weight and tumor volume, respectively (Figures 6B, C), especially in the high-dose group (40 mg/kg). No significant body weight changes were observed, and no mice died during the whole drug administration (data not shown), which indicated drug safety.

## Discussion

The STAT3 SH2 domain is essential for STAT3 dimerization and activity. Strategies to target STAT3 SH2 have been pursued recently by various groups. Several STAT3 inhibitors have been discovered, and some are in clinical trials. STA-21 reportedly exhibited treatment effects against psoriasis and rheumatoid arthritis but remains unclear concerning cancer treatment (Bendell et al., 2014). LLL12 had promising results in preclinical research, however, with low solubility and low bioavailability (Furqan et al., 2013). S3I-201 and its analog S3I-201.1066 were identified by a computational screening assay as a STAT3 SH2 domain inhibitor with IC50 of 86 and 35 μM, respectively (Siddiquee et al., 2007; Fletcher et al., 2009; Zhang et al., 2010; Furqan et al., 2013). S3I-201 shows potent inhibition of STAT3 DNA binding activity with IC50 of 86 ± 33 μM in cell-free assays and low activity toward STAT1 and STAT5 and with STAT3 inhibition of cell lines typically at concentrations from 50 to 100 µM (Siddiquee et al., 2007). Cryptotanshinone is a STAT3 inhibitor with IC50 of 4.6 μM in a cell-free assay and strongly inhibits phosphorylation of STAT3 Tyr705 but is without activity against STAT1 or STAT5. Cryptotanshinone is reported to inhibit STAT3 of cell lines at typical concentrations from 5 to 50 μM (Li et al., 2015). OPB-31121, OPB-51602, and OPB-111077 are STAT3 SH2 domain inhibitors with high binding activity and were investigated by Otsuka Pharmaceutical Company (Lau et al., 2019) but failed clinical trials due to poor pharmacokinetic properties, toxicity, and intolerability (Bendell et al., 2014; Wong et al., 2015). The analog OPB-111077 has demonstrated limited preliminary efficacy, although with better tolerance, in Phase I clinical trial with patients of advanced hepatocellular carcinoma (HCC) (Yoo et al., 2019). C188 and C188-9 were reported to bind directly to STAT3 with high affinity at nanomolar concentrations and showed good oral bioavailability in mice (Bharadwaj et al., 2016), but their efficacy in clinical trials remains unsettled. Mostly, these STAT3 SH2 domain inhibitors showed insufficient potency, poor bioavailability, or selectivity (Johnson et al., 2018; Heppler and Frank, 2019). Therefore, the discovery of novel STAT3 SH2 domain inhibitors abrogating key kinase signaling is a good strategy to augment the drug response. It is becoming more interesting to identify new STAT3 inhibitors due to the unfavorable clinical outcomes of the current STAT3 modulators.

We have shown in this work that two compounds 323–1 and 323–2 were identified as STAT3 SH2 domain inhibitors with better potency than the S3I-201 in terms of drug-target affinity as well as than cryptatanshinone regarding the capability of deactivating STAT3 signaling. Tyr705 phosphorylation, which is vital for STAT3 dimerization and is prevalent in prostate cancer (Horinaga et al., 2005; Liu et al., 2012; Bosch-Barrera and Menendez, 2015), was demonstrated to be downregulated by both compounds, followed by reduced STAT3 transcriptional activity. MTS proliferation and clonogenic assays used on a panel of various tumor cells, including TICs and human prostate cancer cells, indicated that 323s can potently inhibit tumor cell proliferation. DARTS identified that 323s directly bind to STAT3 over STAT1, without affecting SRC or JAK2. Both 323s were predicted to bind to three subpockets of the STAT3 SH2 domain with full inhibition of STAT3, according to the computational docking analyses. Long-time treatment with 323 was required for the 323s to disrupt STAT3 dimerization, which was validated by the co-IP assay within 24 h. In the co-IP assay, the inhibitory effect on STAT3 dimerization was more potent than S3I-201 and consistent with the computational docking data. The FP assay further confirmed that compounds 323–1 and 323–2 target the STAT3 SH2 domain by competitively abrogating the interaction between STAT3 and the phosphopeptide GpYLPQTV with Kd values of 94 and 75 μM, respectively, around five-fold more potently than S3I-201.

However, the short-time 4 h treatment with drugs did not show disruption of STAT3 dimerization in the co-IP assay (Supplementary Figure S8B). One possible reason could be the weak potency of 323s to directly bind to the STAT3 SH2 domain, thereby leading to a weak inhibitory effect on STAT3 dimerization. Another reason could be technical difficulties in the detection of transient and robust interactions in the co-IP assay (De Mol et al., 2018).

The FP assay allows quantitative analysis of molecular interactions based on a single fluorescent label strategy. Fewer proteins and compounds are needed for the experimental steps, and samples might be repetitively measured without being destroyed. These benefits of the FP assay are one important approach in HTS (Lea and Simeonov, 2011). However, the FP assay is sensitive to autofluorescence of ligands, which may confound the sample FP calculation. These interference effects may be flagged by using the kinetic read or background subtraction (Lea and Simeonov 2011). A plate with only peptides and ligands was utilized in parallel to subtract the background and determine Ki values of protein–ligand interaction. Moreover, in the FP assay, it may be hard to detect weak protein–protein interactions due to the limited affinity of the fluorescent ligand (Huang, 2003; Lea and Simeonov, 2011). Another disadvantage relates to the unspecific binding of fluorophores to the protein-binding partner and thus interfere with the final output. Therefore, the DARTS assay was utilized to further validate whether 323–1 and 323–2 are direct STAT3-binding compounds.

According to the FP assay, the ligands 323–1 and 323–2 were found to interrupt the binding between GpYLPQTV and STAT3. Notably, both 323–1 and 323–2 exhibited increased polarization at concentrations above 200 µM. These compounds did not appear to be fluorescent at the measured wavelengths, so at high concentrations, some interaction occurs between the drug and fluorophore to decrease the fluorophore’s mobility. This was confirmed by measuring the polarization of peptides in the presence of high concentrations of the drugs and without STAT3. In particular, at concentrations above 200 µM of 323 drugs, GpYLPQTV became significantly polarized in the absence of STAT3. Another reason is due to the autofluorescence of drugs. The 323 drugs have minor emissions at the wavelengths tested for the GpYLPQTV probe. The emission also appears to be polarized, and the polarization increases with an increasing concentration of 323 drugs in the presence of STAT3. This is further evidence for the binding of the drugs to STAT3.

323s appear to bind to the STAT3 SH2 domain with favorable affinity and selectivity compared with other STAT3 inhibitors, especially S3I-201. Modification of the 323s structure may be executed to get more analogs and improve potency and selectivity. The small molecule-based PROTAC technology is reported to degrade target proteins selectively with high potency *via* the ubiquitin-proteasome mechanism (An and Fu, 2018; Heppler and Frank, 2019; Lau et al., 2019; Pettersson and Crews, 2019). To achieve a higher potency, excellent selectivity, pharmacokinetic properties, and minimal toxicities, we may generate the compound 323–2 or its analog-based STAT3 PROTAC degrader, which directly binds to the STAT3 SH2 domain.

Taken together, our study identified two STAT3 SH2 domain inhibitors, which also provide diversified biological activities and structural diversity for anti-tumor drug discovery and might be promising in the treatment of cancers with hyper-activated STAT3.

## Data Availability

The original contributions presented in the study are included in the article/Supplementary Materials, further inquiries can be directed to the corresponding authors.
