# Supplementary figures and images for: Novel STAT3 Inhibitors Targeting STAT3 Dimerization by Binding to the STAT3 SH2 Domain
Source: Front Pharmacol. 2022 May 27;13:836724. doi: 10.3389/fphar.2022.836724 (PMC9196127; doi:10.3389/fphar.2022.836724)

Figure 1

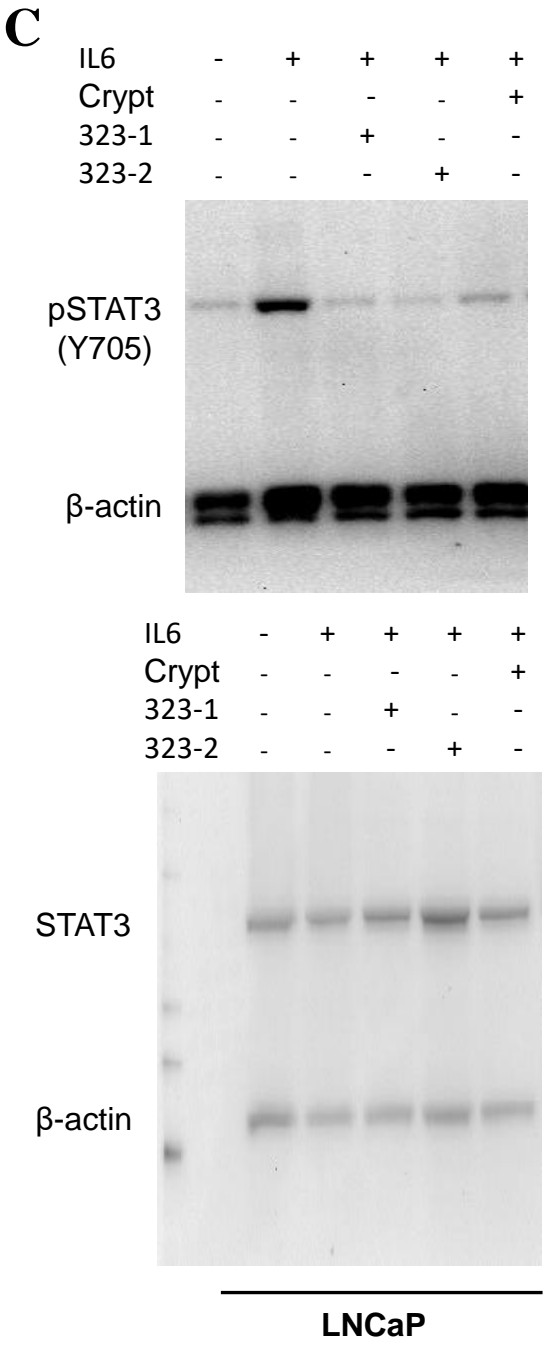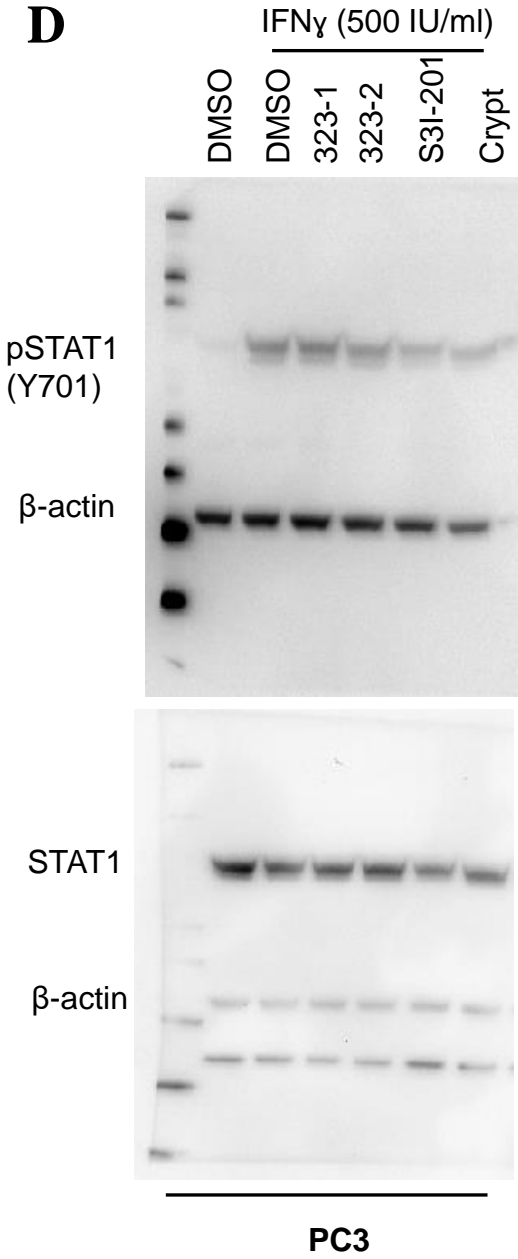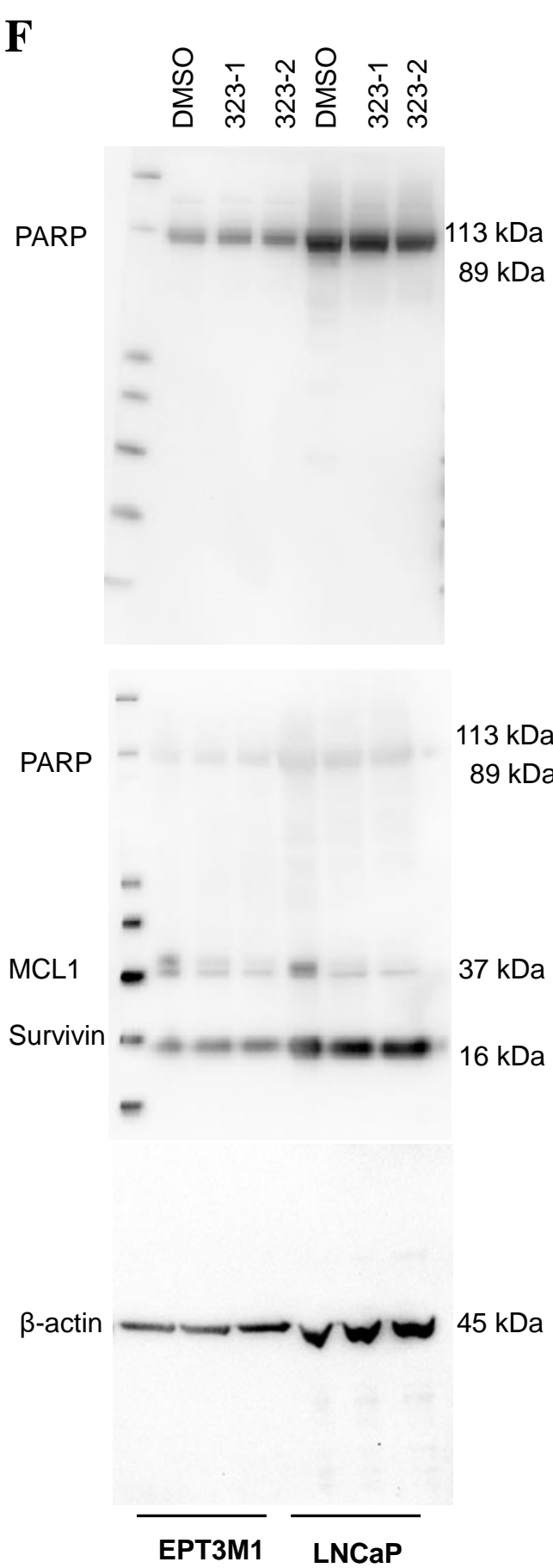

Figure 1E

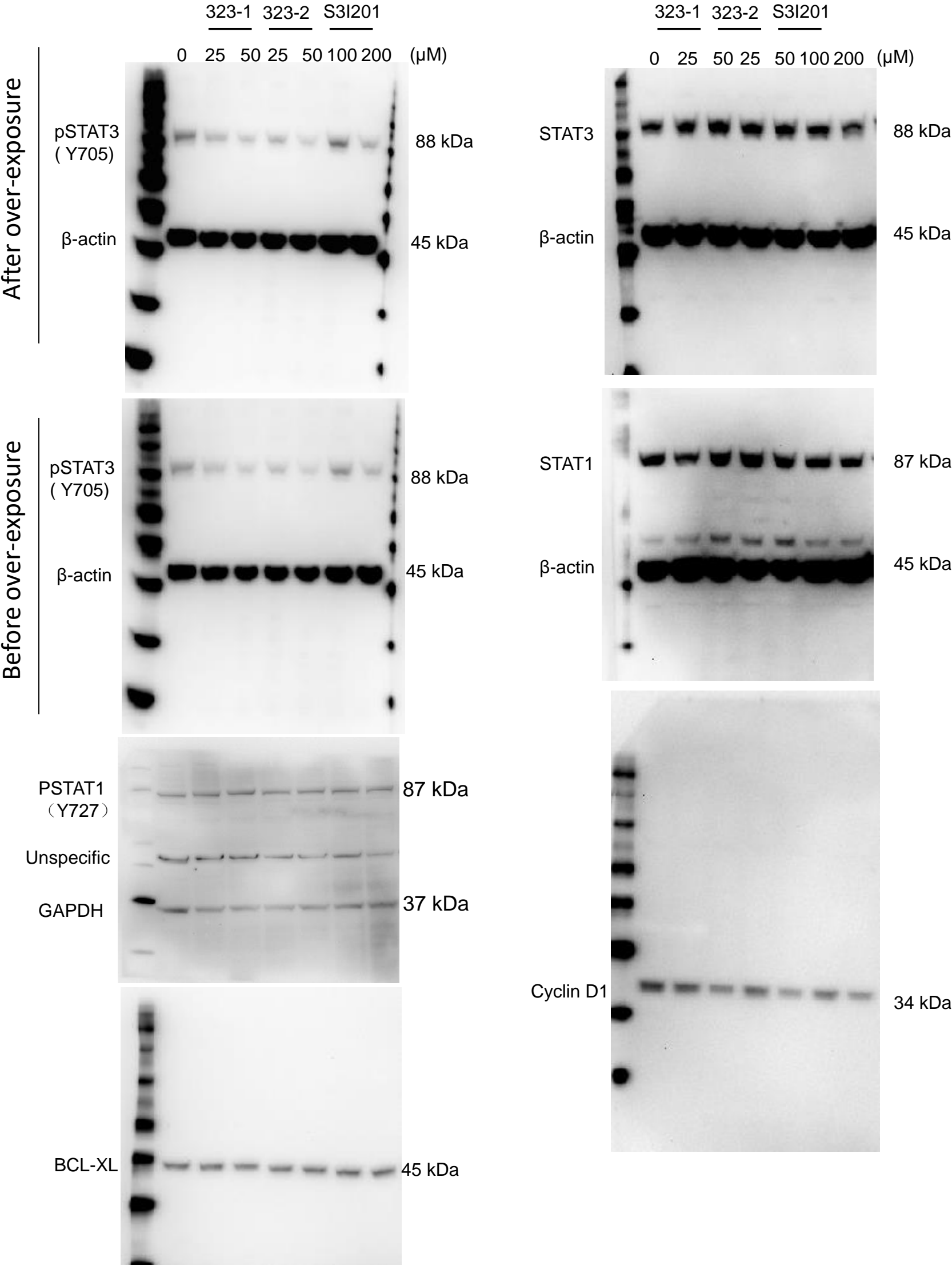

Figure 2B

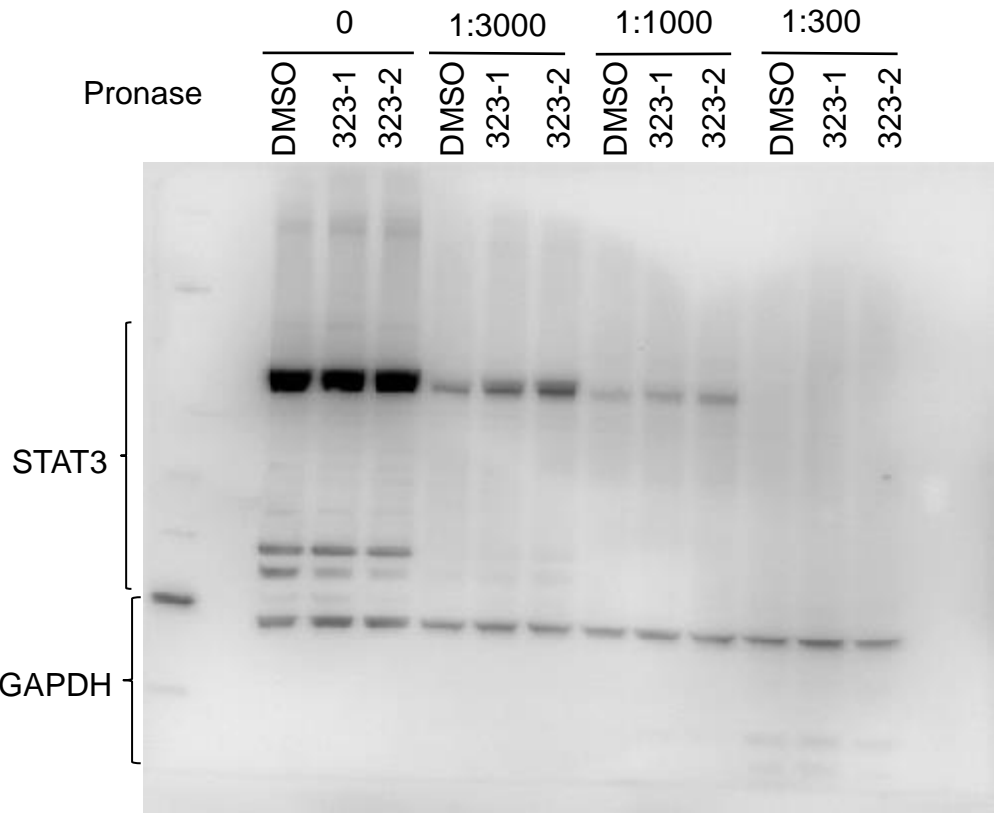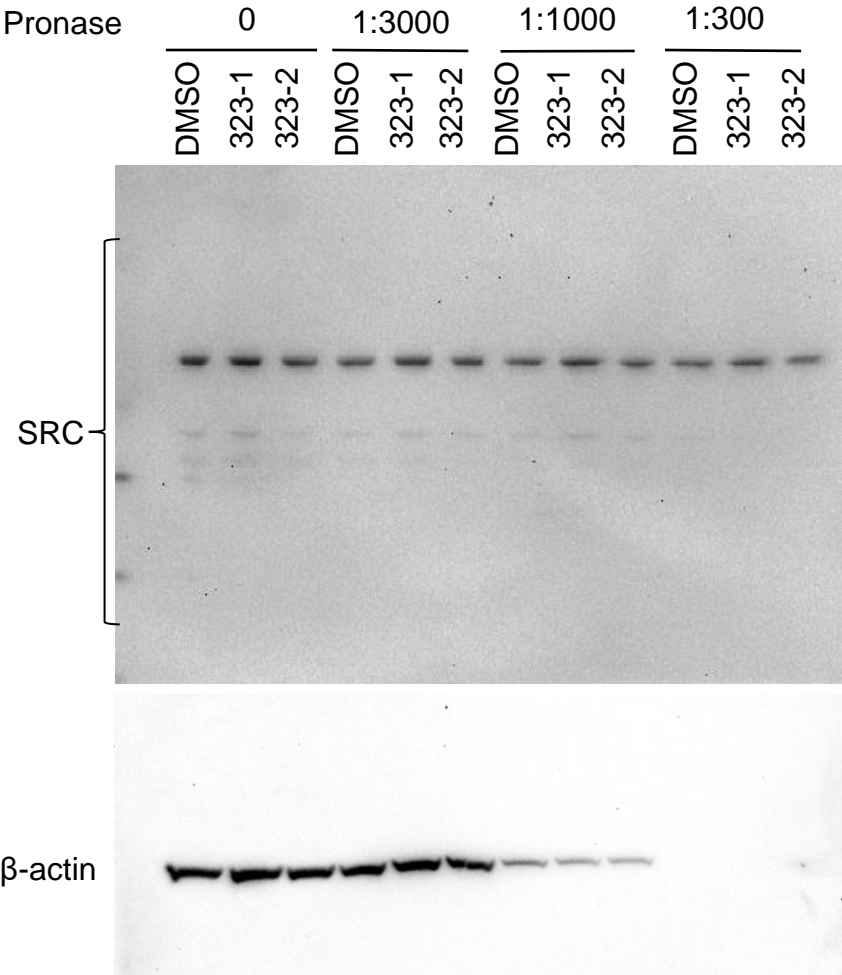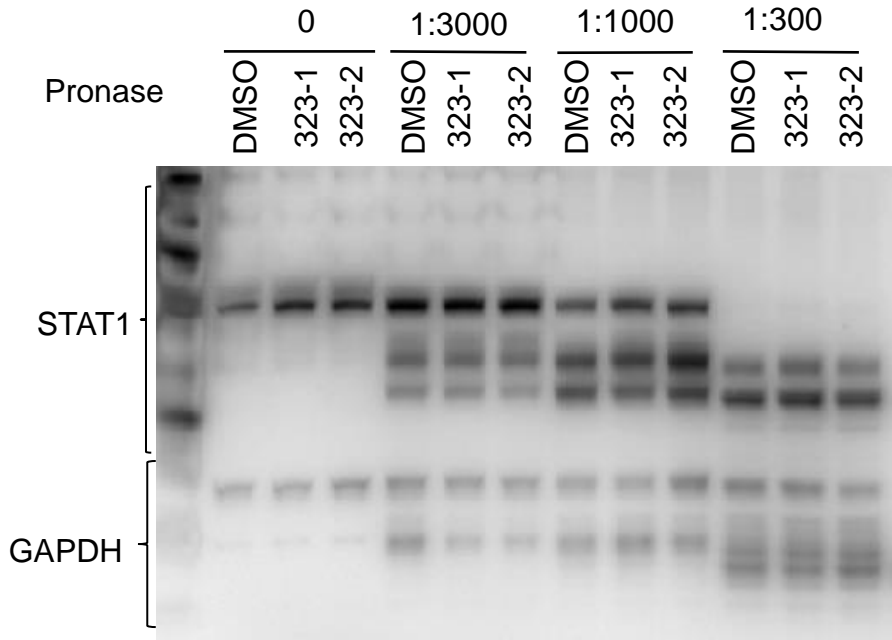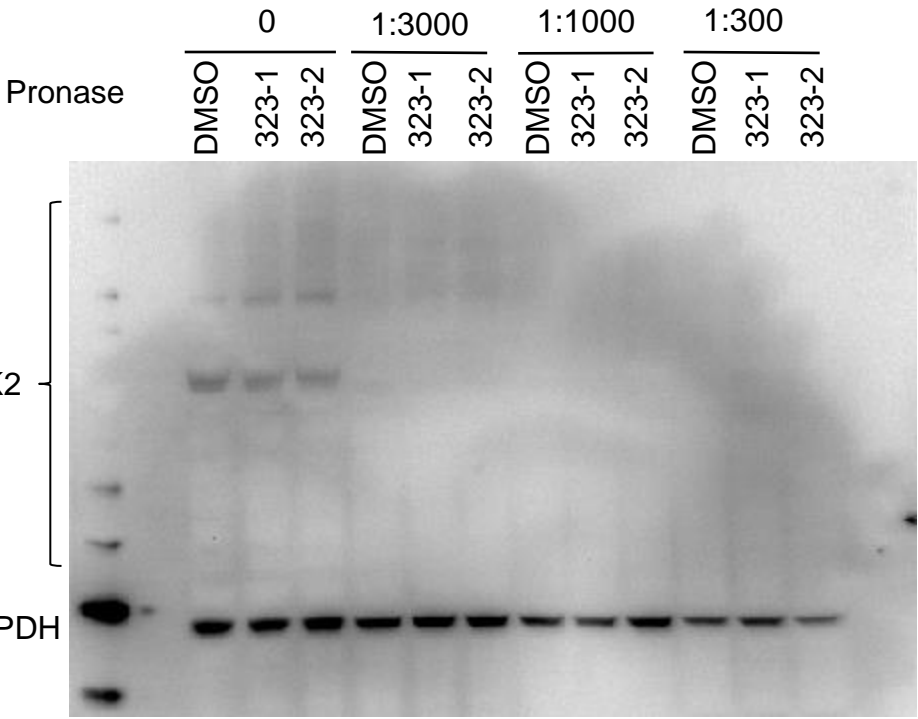

Figure 3A

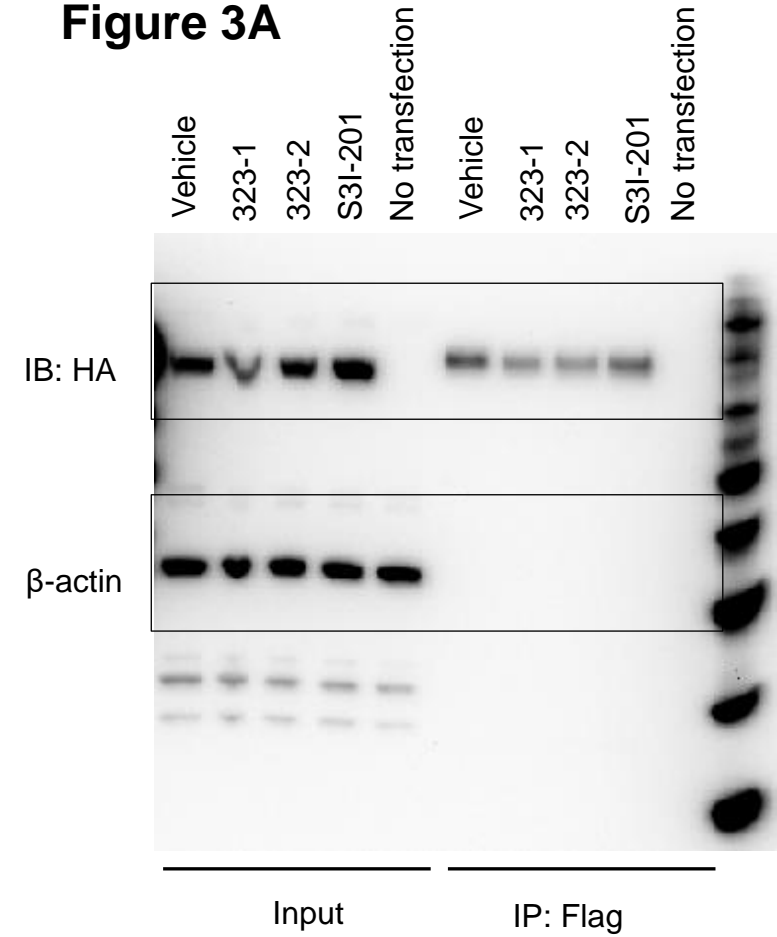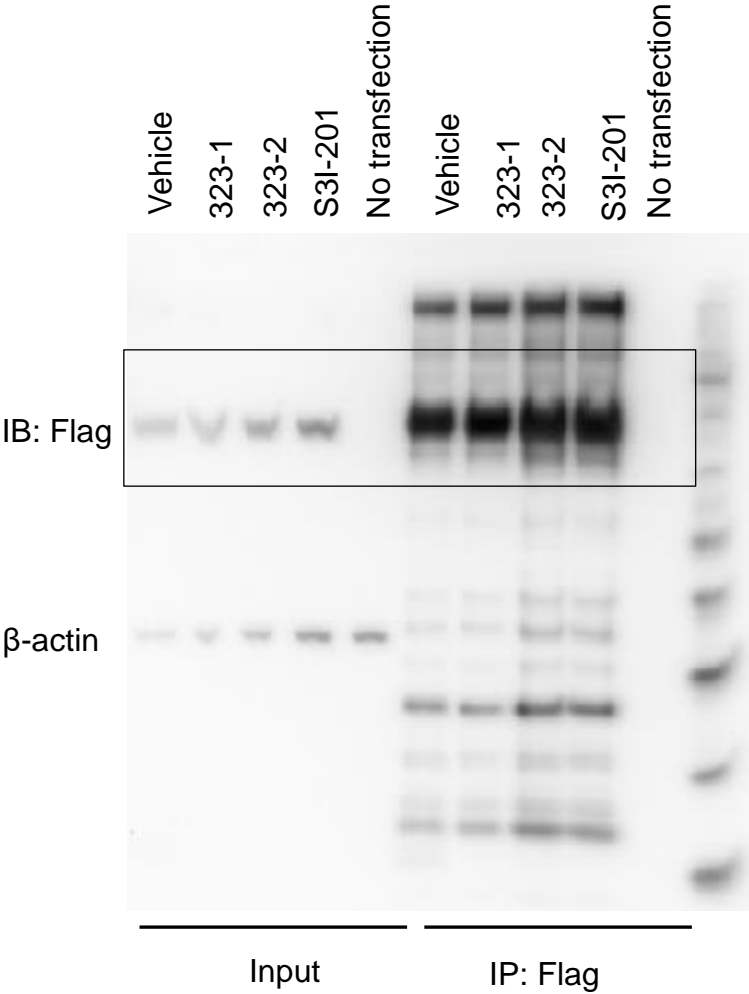

Supplementary Figure 5

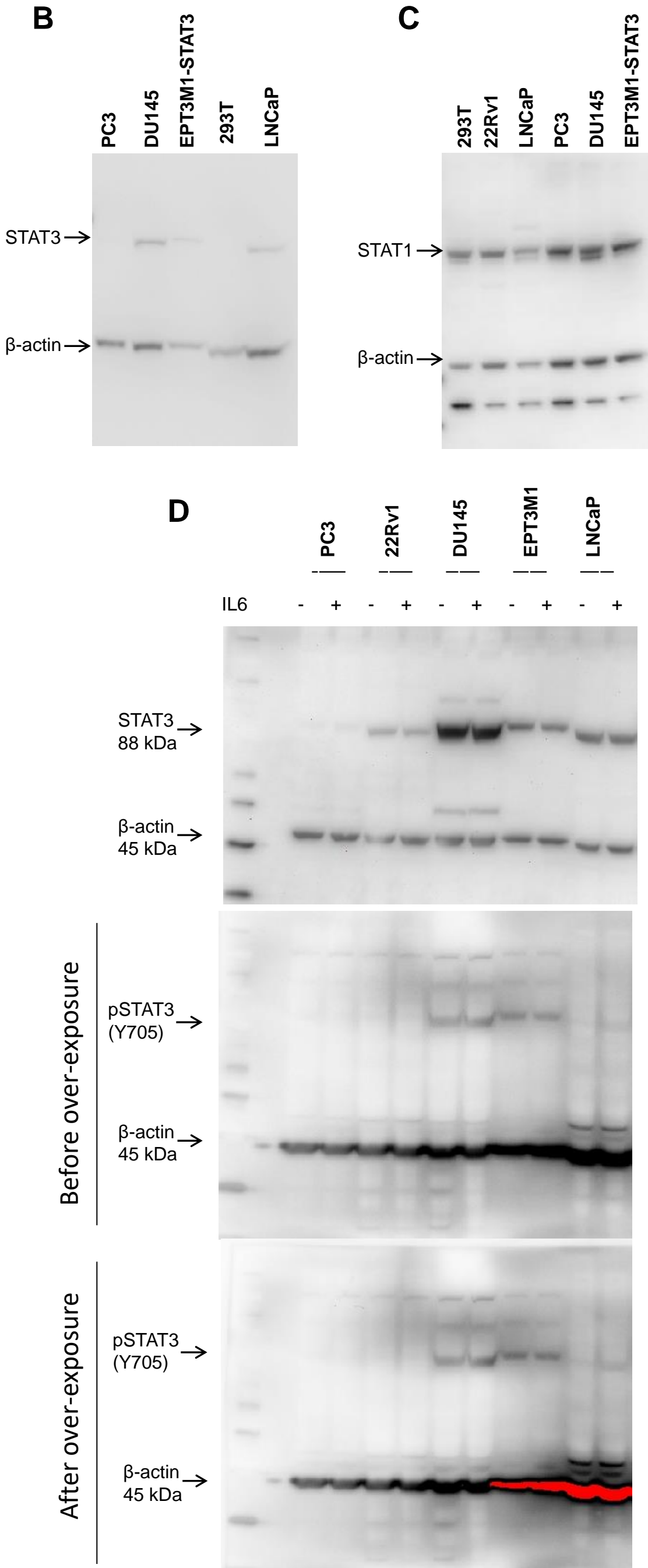

Supplementary Figure 6B

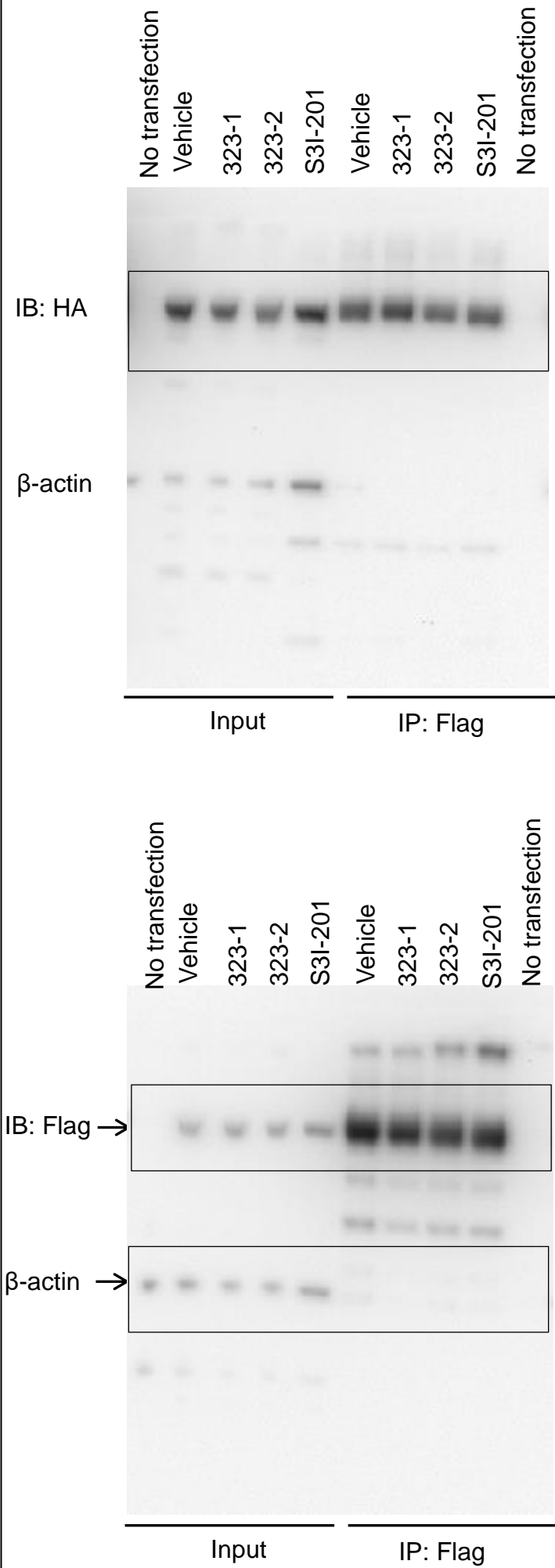

Supplement: Supplementary file 1 [file DataSheet2.pdf]
