# Supplementary material for: Novel STAT3 Inhibitors Targeting STAT3 Dimerization by Binding to the STAT3 SH2 Domain
Source: Front Pharmacol. 2022 May 27;13:836724. doi: 10.3389/fphar.2022.836724 (PMC9196127; doi:10.3389/fphar.2022.836724)
Supplement: Supplementary file 2 [file DataSheet1.pdf]

Supplementary Material

Supplementary Tables

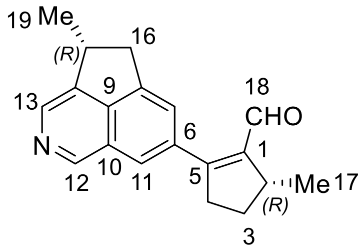

Compound 323-1

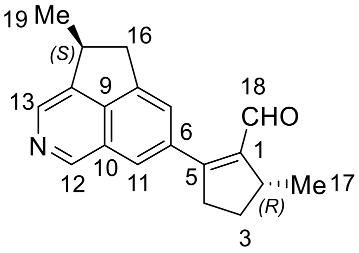

Compound 323-2

Supplementary Table 1. The NMR data of 323-1 and 323-2 ($CDCl_3$, ^1H NMR 400 MHz, ^{13}C NMR 100 MHz)

No.	**323-1**			No.	**323-2**	
	δ_H (*multi.*, *J* in Hz)	δ_C			δ_H (*multi.*, *J* in Hz)	δ_C
1		144.2		1		144.3
2	3.30 (m)	39.1		2	3.30 (m)	39.2
3a	2.28 (m)	30.7		3a	2.28 (m)	30.8
3b	1.67 (m)			3b	1.67 (m)	
4a	3.10 (m)	38.0		4a	3.11 (m)	38.1
4b	2.94 (m)			4b	2.93 (m)	
5		162.2		5		162.3
6		136.1		6		136.2
7	7.42 (brd, 1.13)	123.4		7	7.41 (brd, 1.2)	123.5
8		144.2		8		144.3
9		141.6		9		141.7
10		125.8		10		125.9
11	7.70 (s)	122.3		11	7.69 (brd, 0.65)	122.4
12	9.10 (s)	146.8		12	9.09 (s)	146.8
13	8.46 (s)	137.2		13	8.45 (s)	137.3
14		143.6		14		143.7
15	3.89 (m)	37.3		15	3.88 (m)	37.4
16a	3.68 (dd, 17.6, 7.8)	39.5		16a	3.68 (dd, 17.6, 7.6)	39.6
16b	2.97 (dd, 17.6, 3.3)			16b	2.97 (dd, 17.6, 3.5)	
17	1.50 (d, 7.2)	19.5		17	1.49 (d, 7.1)	19.5
18	9.80 (s)	190.3		18	9.80 (s)	190.3
19	1.24 (d, 6.9)	21.4		19	1.24 (d, 6.9)	21.5

Compounds **323-1** and **323-2** was identified as delavatine A and (15*S*, 2*R*)-delavatine A, respectively, by analysis of NMR spectroscopic data and comparing the NMR data with those reported in literature (Zhang et al., 2017). Moreover, compounds **323-1** and **323-3** have the optical rotations +62.0 (c = 0.05 in $CHCl_3$) and -6.7 (c = 0.05 in CHCl3), respectively. Both compounds were run chiral HPLC chromatography (see Supplementary Figure 5 and Supplementary Figure 6), which exhibited two compounds had different retention times on a chiralpak ID (ID00CD-TB002) column.

Supplementary Table 2. Chiral HPLC parameters

Column	Chiralpak ID (ID00CD-TB002)
Column size	0.46 cm I. D. × 15 cm L
Injection	0.2 μL
Mobile phase	Hexane/EtOH = 50/50 (V/V)
Flow rate	1.0 mL/min
Wavelength	UV 210 nm
Temperature	35 °C
HPLC equipment	Shimadzu LC-20AT
Samples	**323-1**, **323-2**

Optical rotation data

Rudolph Research Analytical

Monday, 09/02/2019

This sample was measured on an Autopol VI, serial number 90079,

manufactured by Rudolph Research Analytical,Hackettstown,NJ.

LotID: 323-1

Set Temperature: 20.0

Temp Corr: OFF

n Average Std.Dev. Maximum Minimum

6 62.000 1.1547 64.000 60.000

S.No Sample ID Time Result Scale OR ₒ Arc WLG Lg.mm Conc. Temp. Comment

1 323-P1 07:19:15 PM 64.000 SR 0.032 589 100.00 0.050 19.7

2 323-P1 07:19:25 PM 62.000 SR 0.031 589 100.00 0.050 19.7

3 323-P1 07:19:35 PM 60.000 SR 0.030 589 100.00 0.050 19.7

4 323-P1 07:19:45 PM 62.000 SR 0.031 589 100.00 0.050 19.8

5 323-P1 07:19:55 PM 62.000 SR 0.031 589 100.00 0.050 19.8

6 323-P1 07:20:05 PM 62.000 SR 0.031 589 100.00 0.050 19.8

Rudolph Research Analytical

Monday, 09/02/2019

This sample was measured on an Autopol VI, serial number 90079,

manufactured by Rudolph Research Analytical,Hackettstown,NJ.

LotID: 323-2

Set Temperature: 20.0

Temp Corr: OFF

n Average Std.Dev. Maximum Minimum

6 -6.667 1.4907 -4.000 -8.000

S.No Sample ID Time Result Scale OR o Arc WLG Lg.mm Conc. Temp. Comment

1 323-P2 07:29:31 PM -6.000 SR -0.003 589 100.00 0.050 20.2

2 323-P2 07:29:41 PM -8.000 SR -0.004 589 100.00 0.050 20.1

3 323-P2 07:29:56 PM -8.000 SR -0.004 589 100.00 0.050 20.1

4 323-P2 07:30:06 PM -4.000 SR -0.002 589 100.00 0.050 20.0

5 323-P2 07:30:16 PM -6.000 SR -0.003 589 100.00 0.050 20.0

6 323-P2 07:30:26 PM -8.000 SR -0.004 589 100.00 0.050 20.0

Supplementary Figures

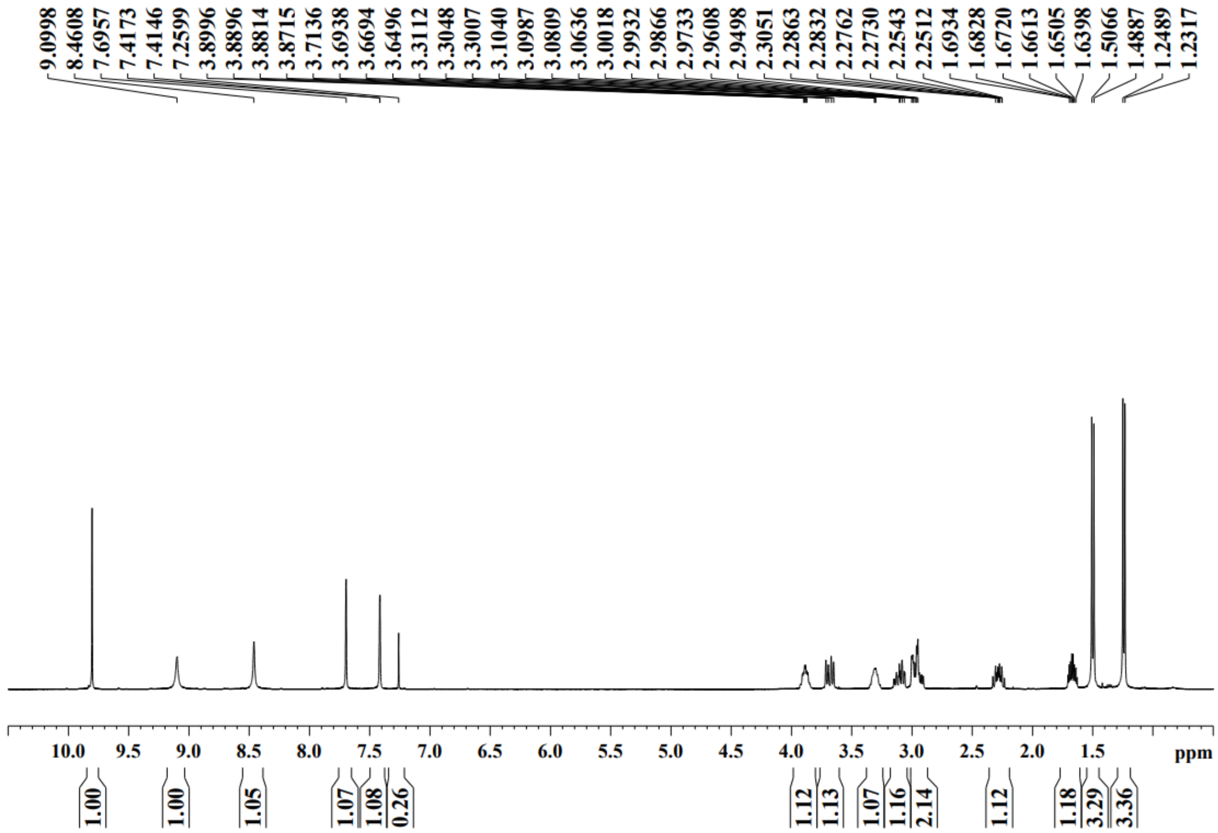

Supplementary Figure 1. The NMR data of 323-1 (CDCl3, 1H NMR 400 MHz)

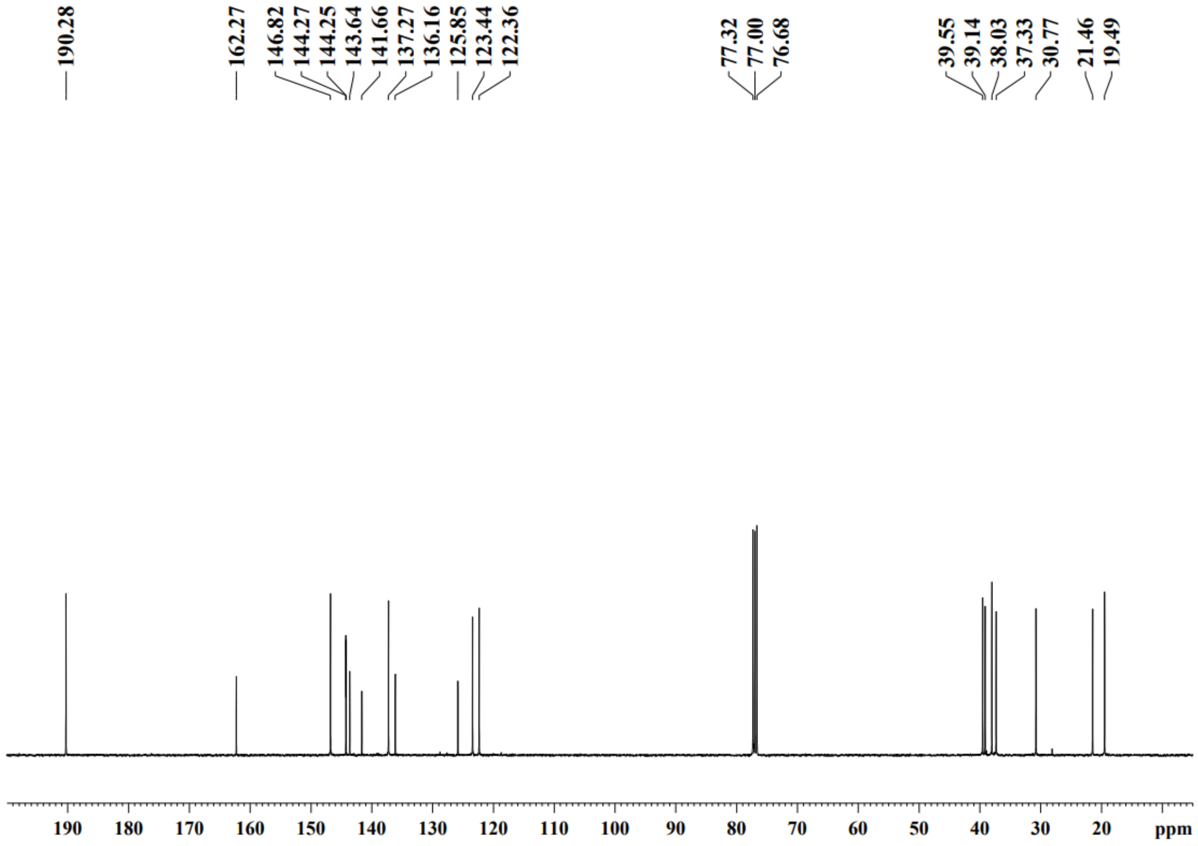

Supplementary Figure 2. The NMR data of 323-1 (CDCl3, 13C NMR 100 MHz).

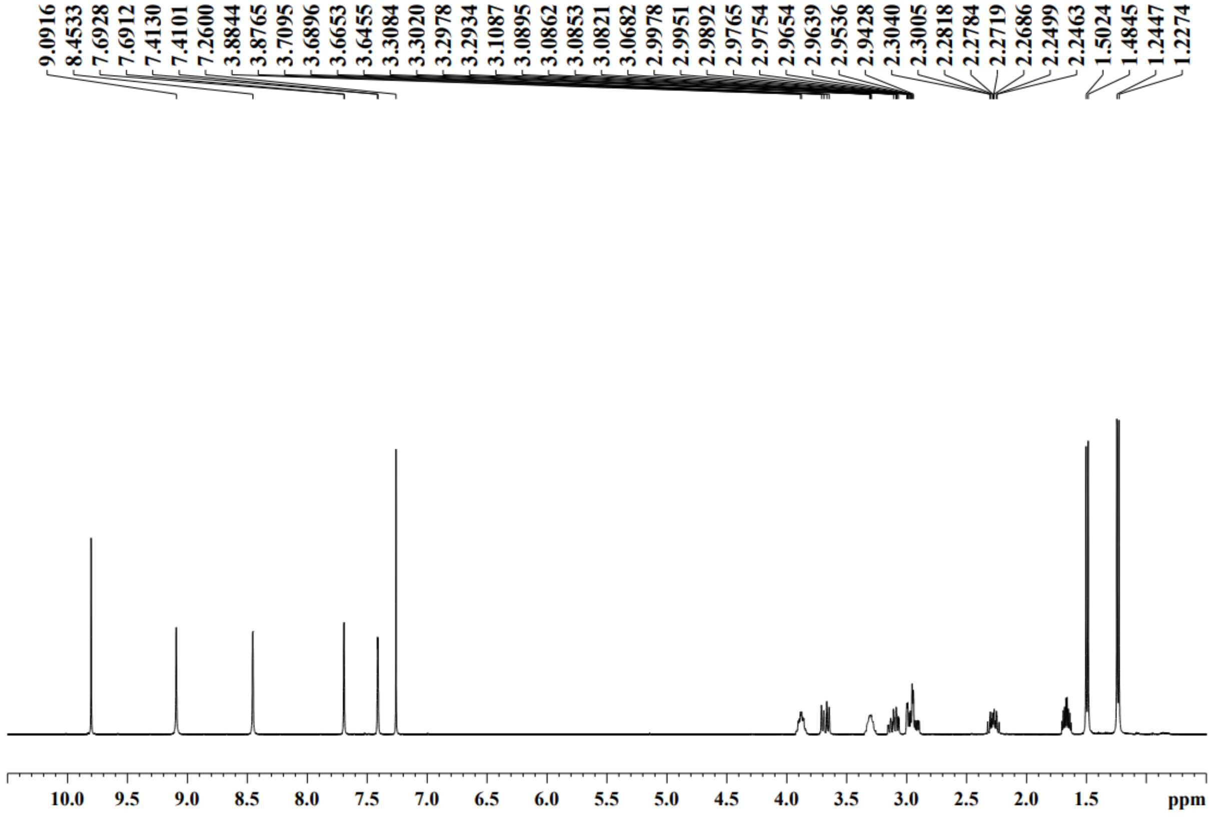

Supplementary Figure 3. The NMR data of 323-2 (CDCl3, 1H NMR 400 MHz).

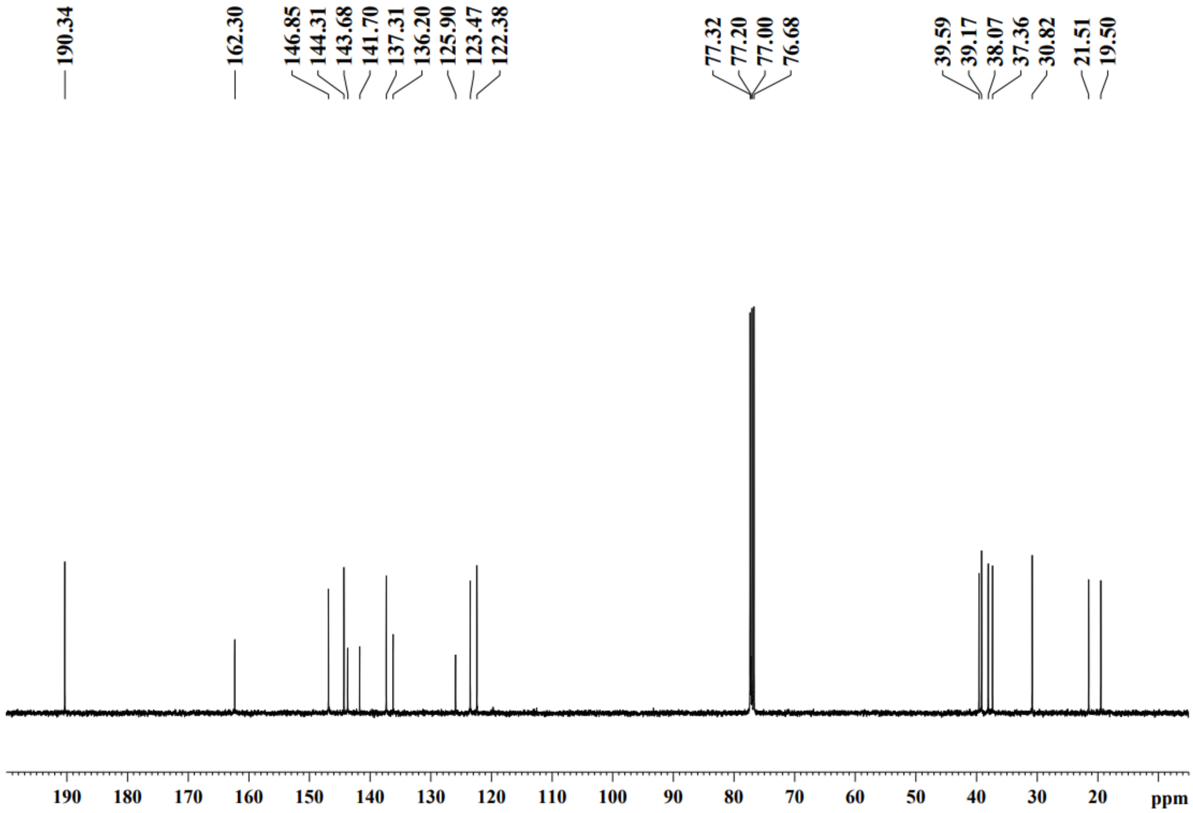

Supplementary Figure 4. The NMR data of 323-2 (CDCl3, 13C NMR 100 MHz).

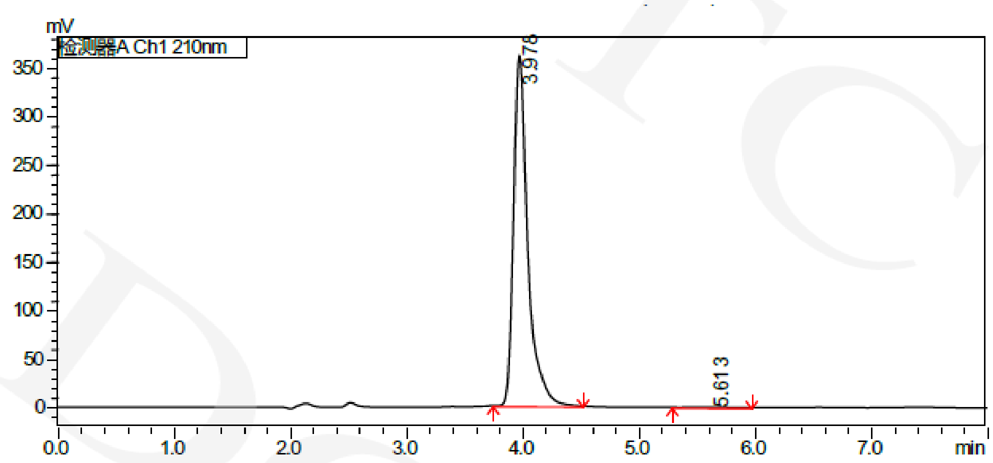

<Peak Table>

Peak#	Ret. Time	Area	Area%	T.Plate#	Tailing F.	Resolution
1	3.978	3075993	99.848	5371	1.583	--
2	5.613	4686	0.152	5286	1.011	6.217

Supplementary Figure 5. The chiral HPLC chromatogram of 323-1

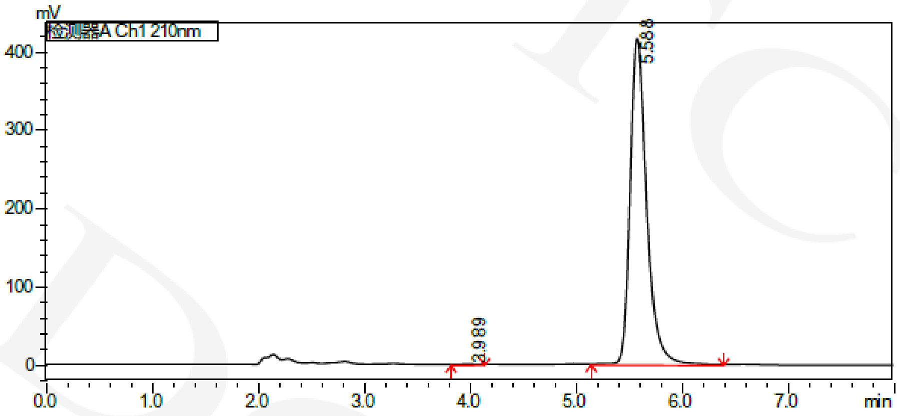

<Peak Table>

Peak#	Ret. Time	Area	Area%	T.Plate#	Tailing F.	Resolution
1	3.989	4732	0.105	4902	0.941	--
2	5.588	4519077	99.895	6258	1.344	6.266

Supplementary Figure 6. The chiral HPLC chromatogram of 323-2

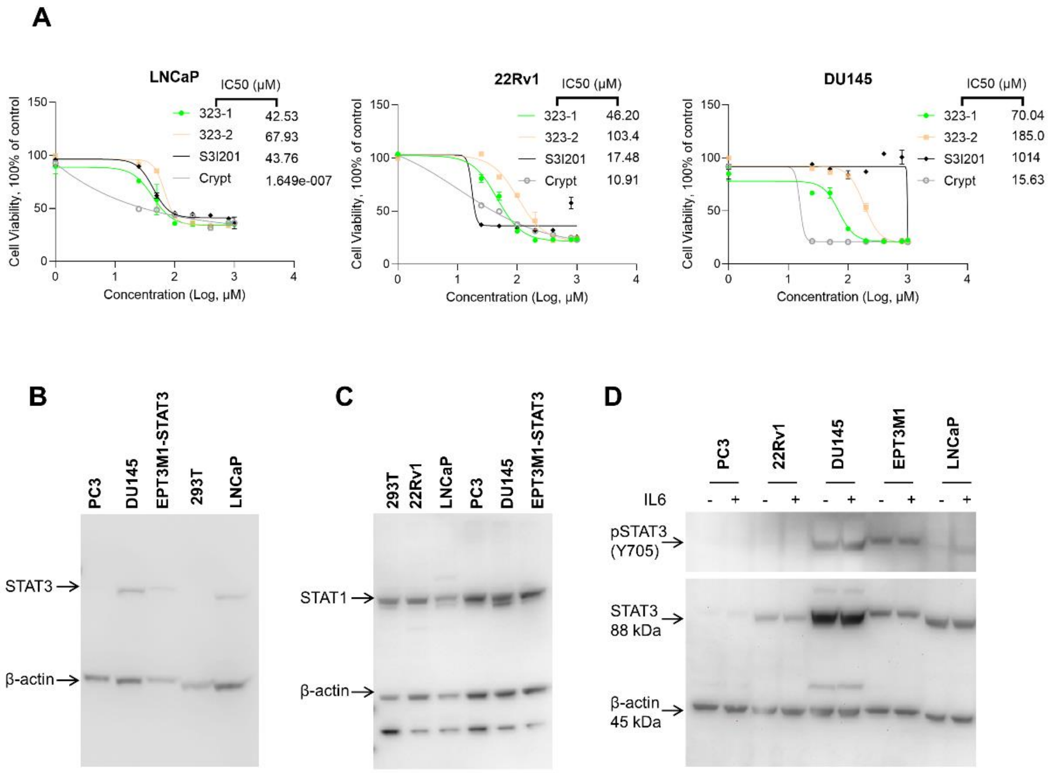

Supplementary Figure 7. 323-1 and 323-2 inhibit cell proliferation/ comparison of STAT3 and STAT1 expression in different cell lines

A) Different cell lines were treated with indicated doses of drugs for 96 hrs. All data are represented as the average ± s.e.m (n=3). Data was analyzed by using Prism software-log(inhibitor) vs. response -- Variable slope (four parameters) with formula Y=Bottom + (Top-

Bottom)/(1+10^((LogIC50-X) *HillSlope)), X: log of dose or concentration, Y: Response, decreasing as X increases, Top and Bottom: Plateaus in same units as Y.

B) Lysates of cell lines were examined using Western blotting with anti-STAT3 antibody.

C) Lysates of cell lines were examined using Western blotting with anti-STAT1 antibody.

D) Various cell lines were treated with 10 ng/ml IL-6 for 15 min and lysates were examined using Western blotting with indicated antibodies.

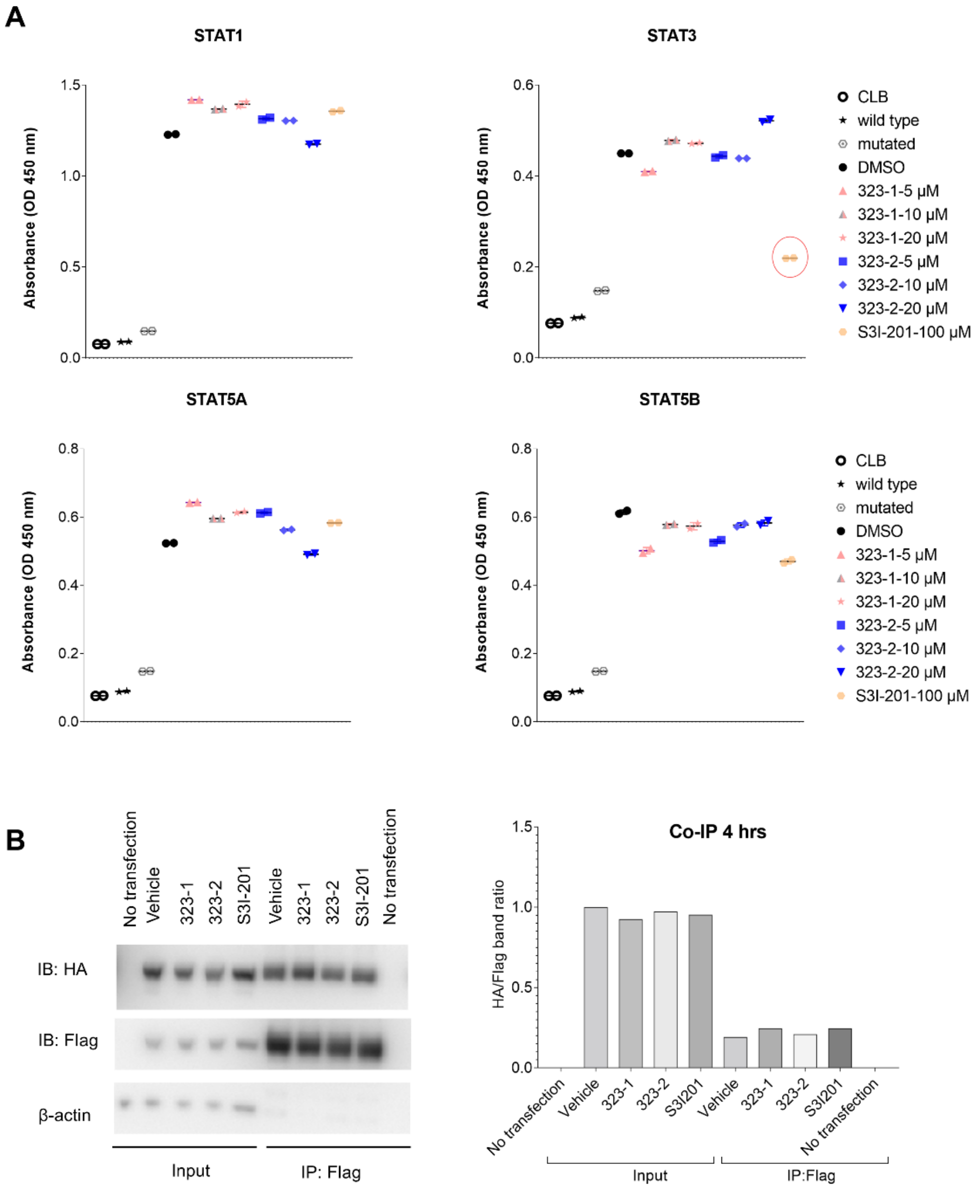

Supplementary Figure 8. 323-1 and 323-2 did not bind to the STAT3 DBD

A) DU145 cells were seeded and treated with 5-20 μM 323-1 or 323-2 for 24 hrs. Then nuclear proteins were extracted from the cell lysate for DNA binding detection to STAT1, STAT3 and STAT5 oligonucleotides according to the protocol as described in the Materials and Method. Complete Lysis Buffer (CLB) was set as blank. Nb2 nuclear extract (Prolactin stimulated) was added with wild-type oligonucleotide (wild-type, competes with sample nuclear extracts for STAT) or the mutated oligonucleotide (mutated, no effect on ability of sample nuclear extracts

to bind to STAT) into the pre-coated 96- well with STAT oligonucleotide as a control to monitor the specificity of the assay. Data are shown as the representative result from three performed.

B) The short-time treatment with 323s did not impair STAT3 dimerization. HEK 293T cells were transfected with FLAG-STAT3 and HA-STAT3 cells were treated with DMSO, 50 μM 323-1, 50 μM 323-2, 200 μM S3I-201 for 4 hrs. Extracts were immunoblotted with anti-HA, anti-FLAG, and anti-β-actin (ab8226). HA/Flag ratio was calculated by using the ImageJ software and Vehicle within Input group was set as control. Data are shown as the representative result of two experiments.
